# Supplementary material for: High-resolution cryo-EM of the human CDK-activating kinase for structure-based drug design
Source: Nat Commun. 2024 Mar 13;15:2265. doi: 10.1038/s41467-024-46375-9 (PMC10937634; doi:10.1038/s41467-024-46375-9)
Supplement: Supplementary file 1 — Supplementary Information [file 41467_2024_46375_MOESM1_ESM.pdf]

Supplementary Information

High-resolution cryo-electron microscopy of the human CDK-activating kinase for structure-based drug design

Victoria I. Cushing, Adrian F. Koh, Junjie Feng, Kaste Jurgaityte, Alexander Bondke, Sebastian H. B. Kroll, Marion Barbazanges, Bodo Scheiper, Ash K. Bahl, Anthony G. M. Barrett, Simak Ali*, Abhay Kotecha*, Basil J. Greber*

* Correspondence should be addressed to:
basil.greber@icr.ac.uk; abhay.kotecha@thermofisher.com; simak.ali@imperial.ac.uk

This file contains:

Supplementary Figures 1-17
Supplementary Tables 1-11
Supplementary Notes 1-2
Supplementary References 1-19

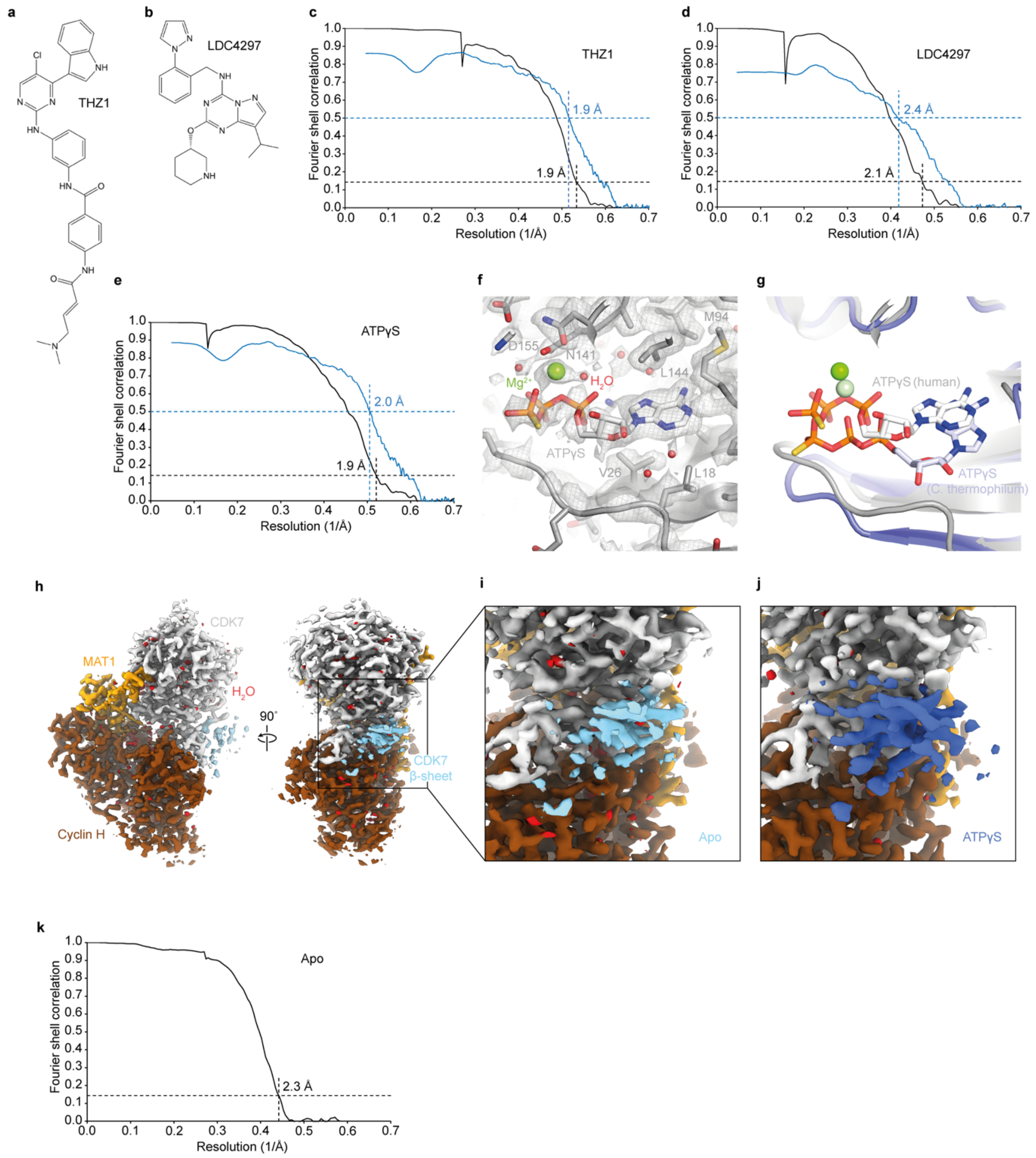

Supplementary Figure 1 | Proof of principle for high-resolution CAK structure determination and analysis of the structures of apo-CAK and CAK with bound nucleotide. (**a**) Chemical structure of the phenylaminopyrimidine-class inhibitor THZ1 that uses a reactive acrylamide group for covalent modification of target kinases. (**b**) Chemical structure of the pyrazolotriazine-class inhibitor LDC4297. (**c**-**e**) Fourier shell correlation (FSC) curves for the CAK-ATPγS, CAK-THZ1, and CAK-LDC4297 cryo-EM reconstructions. Half-map FSC curves are shown in black, model vs. map FSC curves in blue. Resolutions are estimated according to the FSC = 0.143 criterion for half-maps, and the FSC = 0.5 criterion for model vs. map FSCs[1]. (**f**) Structure of the ATPγS-bound active site of CAK shown with the cryo-EM map. (**g**) ATPγS molecule bound in the active site of human CDK7 (white) compared to adenosine nucleotide

bound in the active site of fungal CDK7 (PDB ID 6Z4X; light blue). (**h**) Cryo-EM map of apo-CAK, with β-sheet in the N-terminal kinase lobe shown in cyan. (**i**, **j**) Comparison of the density for the β-sheet in the N-terminal kinase lobe in nucleotide-bound and apo-CAK (domain highlighted in cyan and blue, respectively; CAK-ATPγS map low-pass filtered to 2.3 Å resolution). (**k**) FSC curve for the apo-CAK cryo-EM reconstruction. Source data are provided as a Source Data file.

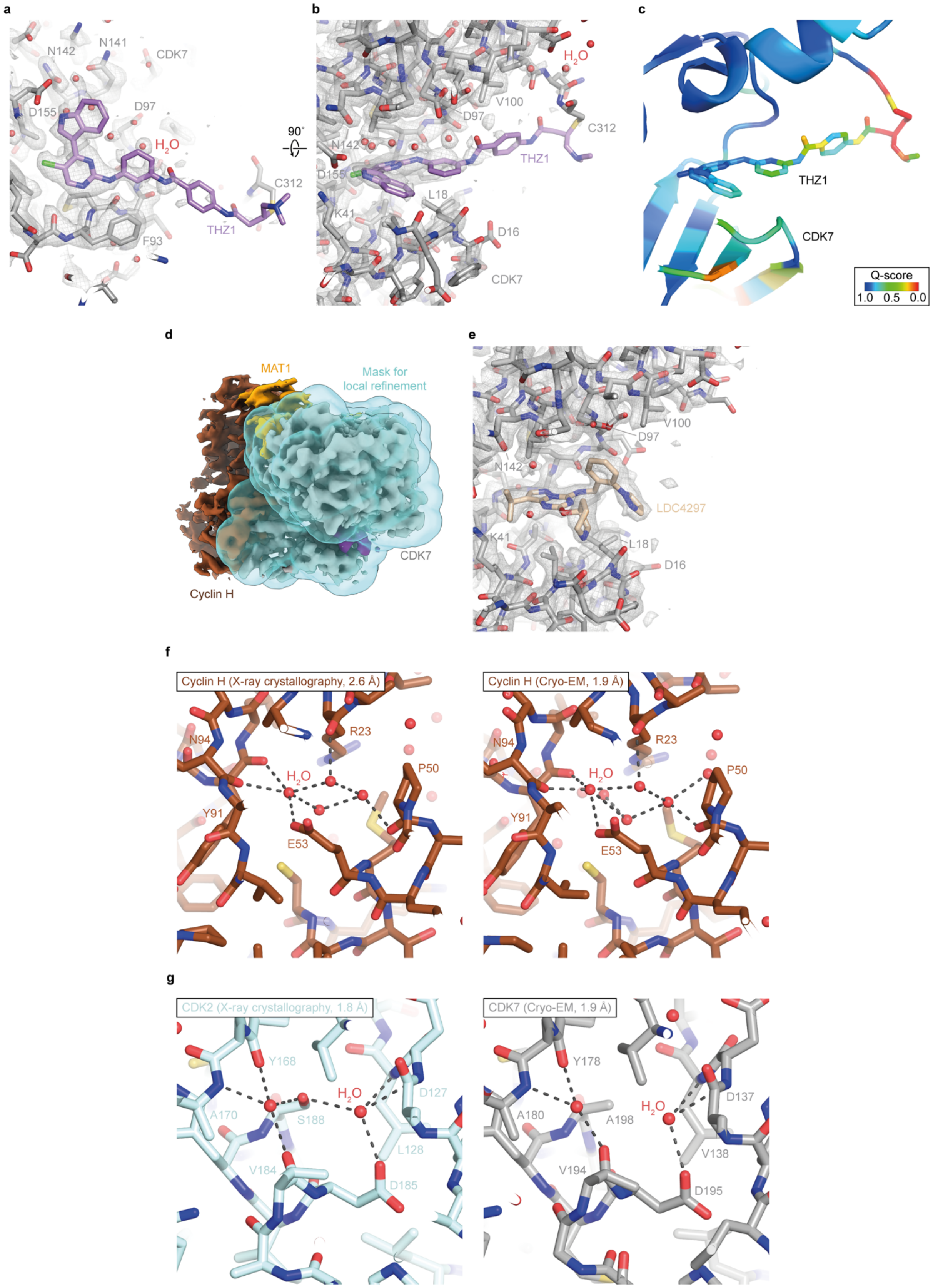

Supplementary Figure 2 | Analysis of the structures of CAK-THZ1 and CAK-LDC4297. (**a**, **b**) The head group of THZ1 occupies the active site of human CDK7, while the cysteine-reactive portion of the inhibitor protrudes from the active site pocket. The cryo-EM map quality is high for the tightly bound portions of the inhibitor and becomes very weak for the less-tightly

bound components of the inhibitor, indicating structural heterogeneity. (**c**) The variable quality of the THZ1 density is quantified using Q-scores. (**d**) Mask used for masked refinement of a 40 kDa-fragment shown in cyan, CDK7 in grey, MAT1 in orange, and cyclin H in brown. (**e**) Anti-viral compound LDC4297 bound in the active site of CDK7. (**f**, **g**) Comparison of locations of bound water molecules in X-ray crystal structures of cyclin H (PDB ID 1JKW; 2.6 Å resolution)[2] and CDK2 (PDB ID 6ATH; 1.8 Å resolution)[3] with the water positions identified in our cryo-EM maps for cyclin H and CDK7 (CAK-THZ1 model used).

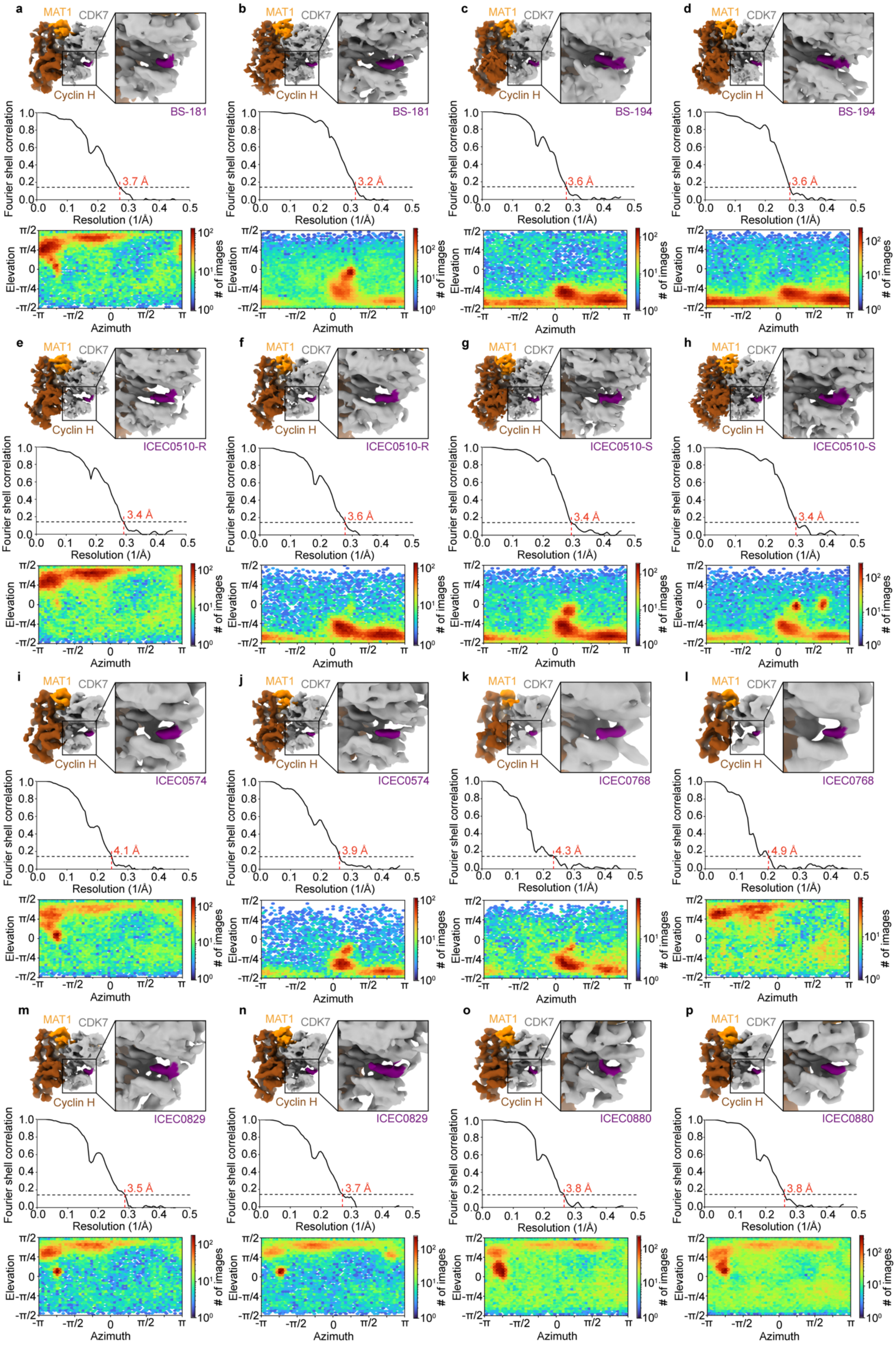
a
MAT1
CDK7
Cyclin H
BS-181
3.7 Å
b
BS-181
3.2 Å
c
BS-194
3.6 Å
d
BS-194
3.6 Å
e
ICEC0510-R
3.4 Å
f
ICEC0510-R
3.6 Å
g
ICEC0510-S
3.4 Å
h
ICEC0510-S
3.4 Å
i
ICEC0574
4.1 Å
j
ICEC0574
3.9 Å
k
ICEC0768
4.3 Å
l
ICEC0768
4.9 Å
m
ICEC0829
3.5 Å
n
ICEC0829
3.7 Å
o
ICEC0880
3.8 Å
p
ICEC0880
3.8 Å
Fourier shell correlation
Resolution (1/Å)
Elevation
Azimuth
# of images

Supplementary Figure 3 | Live processing of 1-hour Glacios datasets, part 1. (**a**-**p**) Overview of 1-hour Glacios datasets. Top panels show a view of the 3D reconstruction (CDK7 grey, cyclin H brown, MAT1 orange) and a close-up view of the density for bound inhibitors (purple). Middle panels show the resolution according to the FSC = 0.143 threshold[1]. Bottom panels show orientation distribution plots from cryoSPARC streaming refinement. Source data are provided as a Source Data file.

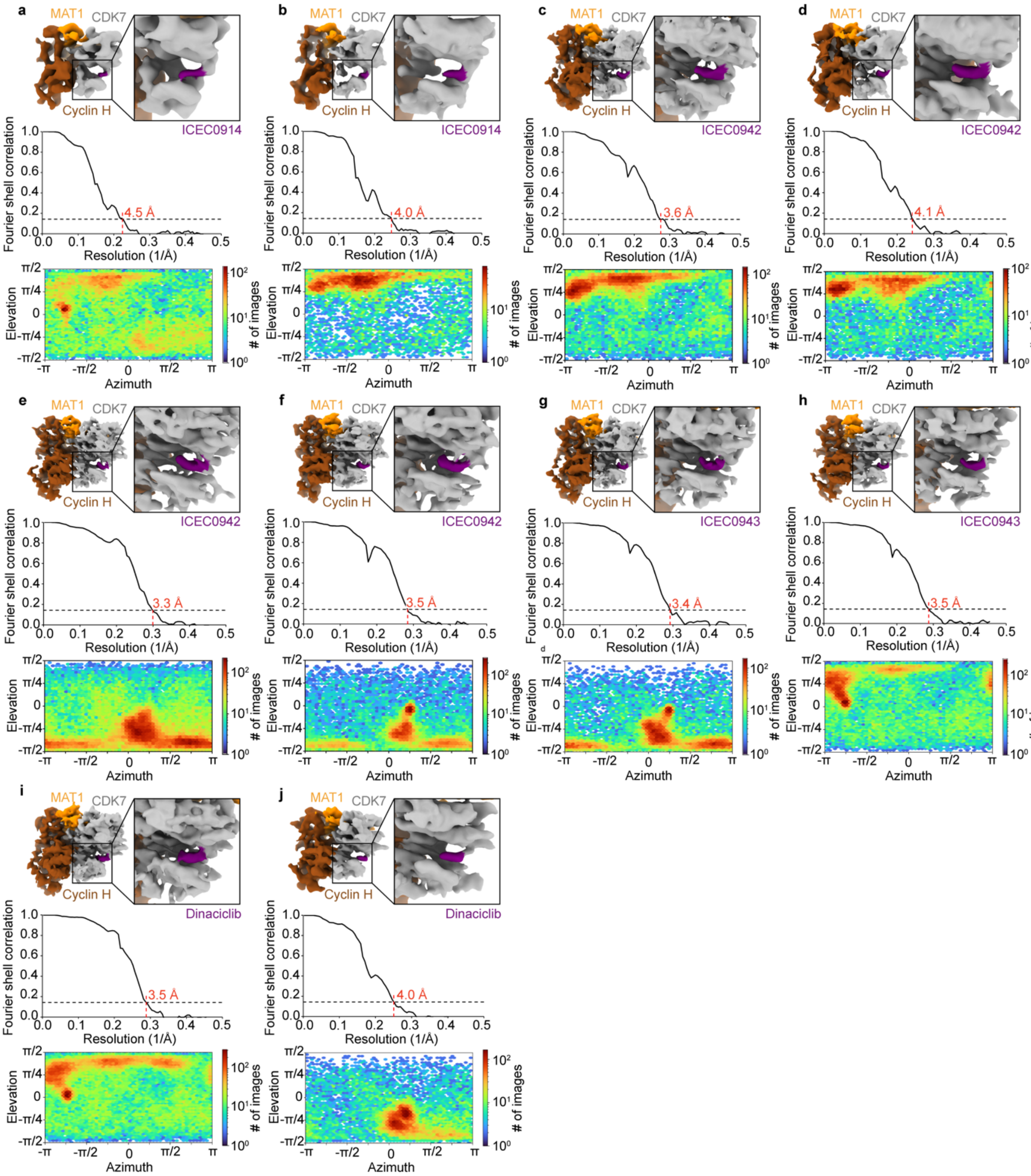

Supplementary Figure 4 | Live processing of 1-hour Glacios datasets, part 2. (**a-j**) Overview of 1-hour Glacios datasets. Top panels show a view of the 3D reconstruction (CDK7 grey, cyclin H brown, MAT1 orange) and a close-up view of the density for bound inhibitors (purple). Middle panels show the resolution according to the FSC = 0.143 threshold[1]. Bottom panels show orientation distribution plots from cryoSPARC streaming refinement. Source data are provided as a Source Data file.

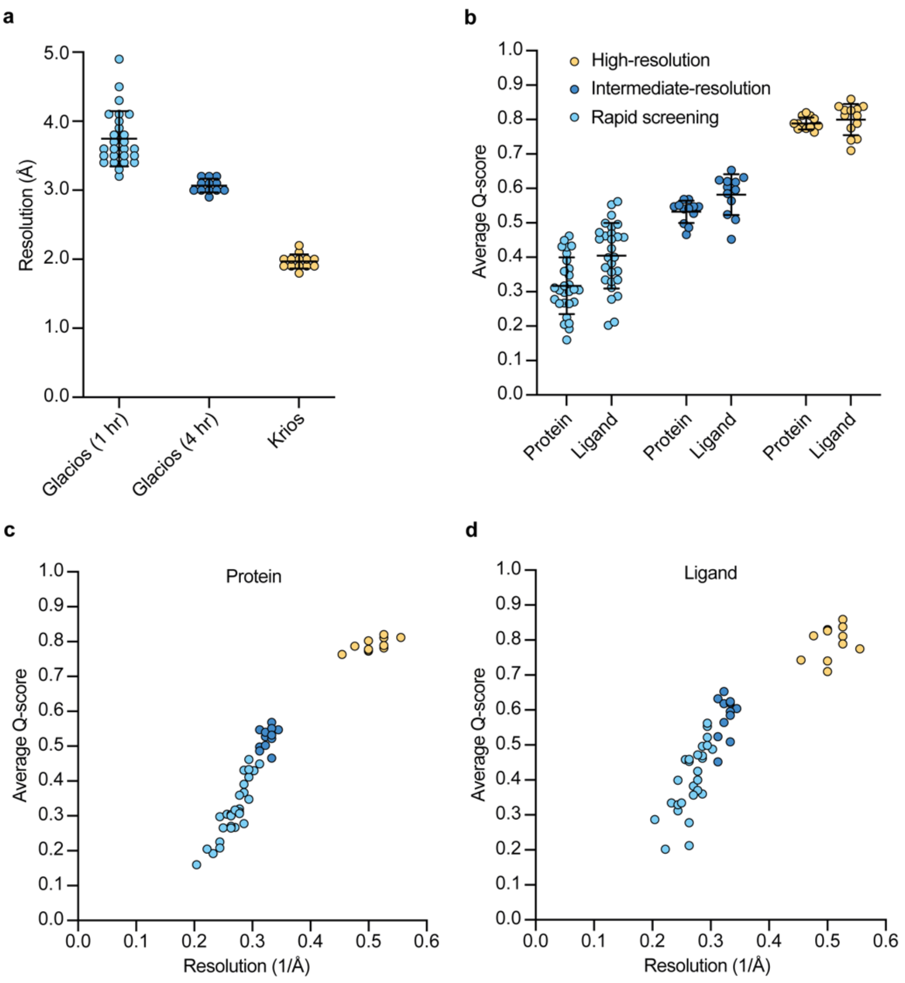

Supplementary Figure 5 | Resolutions achieved and quantitative analysis of map-model fit using Q-scores. (**a**) Resolutions achieved for the three phases of our screening and high-resolution data collection workflow. Mean and standard deviation are indicated. (**b**) Quantitative analysis of map-model fit for protein and ligand densities for the three phases of our workflow. Mean and standard deviation are indicated. Q-scores[4] for the 1-hour and 4-hour screening output maps were calculated against the high-resolution structures, which serve as a good approximation of the ground truth for the lower-resolution maps. (**c**, **d**) Resolution-dependent improvement of Q-scores for protein (c) and ligand (d) densities in datasets collected using our workflow. The number of data points for the graphs corresponds to the number of structures obtained at each screening stage. Source data are provided as a Source Data file.

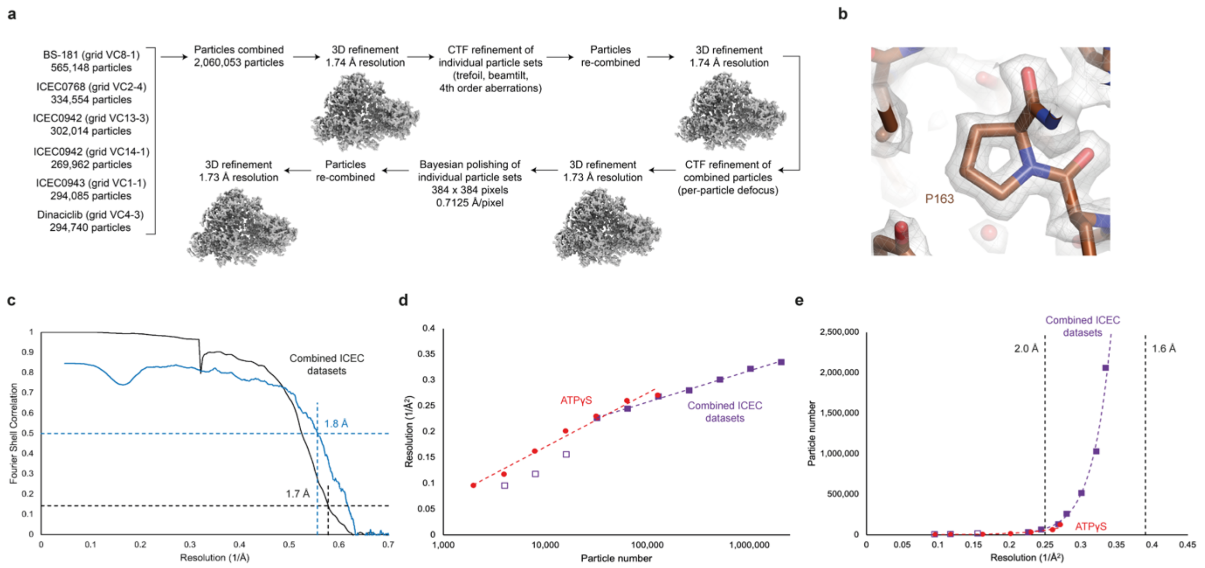

Supplementary Figure 6 | The 1.7 Å-resolution cryo-EM structure of the human CAK. (**a**) High-resolution processing workflow leading to a 1.7 Å-reconstruction of the human CAK with averaged inhibitor density. (**b**) View of a proline residue (P163 of cyclin H) in the 1.7 Å-resolution reconstruction of the human CAK. (**c**) FSC curves for the 1.7 Å-reconstruction. The half-map FSC is shown in black, the model vs. map FSC is shown in blue. Resolution values are estimated from the FSC = 0.143 criterion for half-maps, and the FSC = 0.5 criterion for model vs. map FSCs [1]. (**d**) Henderson-Rosenthal Plot for particle subsets derived by splitting the 1.7 Å-dataset in half 9 consecutive times (purple). At low particle numbers, the data deviate from linearity (outlined purple squares). These points were not used for extrapolation to higher particle numbers (see panel e). Additionally, data for the ATPγS dataset, which reached higher resolutions at low particle numbers, possibly due to the absence of DMSO in the sample buffer, is plotted in red. (**e**) The data from panel d plotted with particles on a linear scale to visualize the exponential increase in particle number required to reach higher resolution. Source data are provided as a Source Data file.

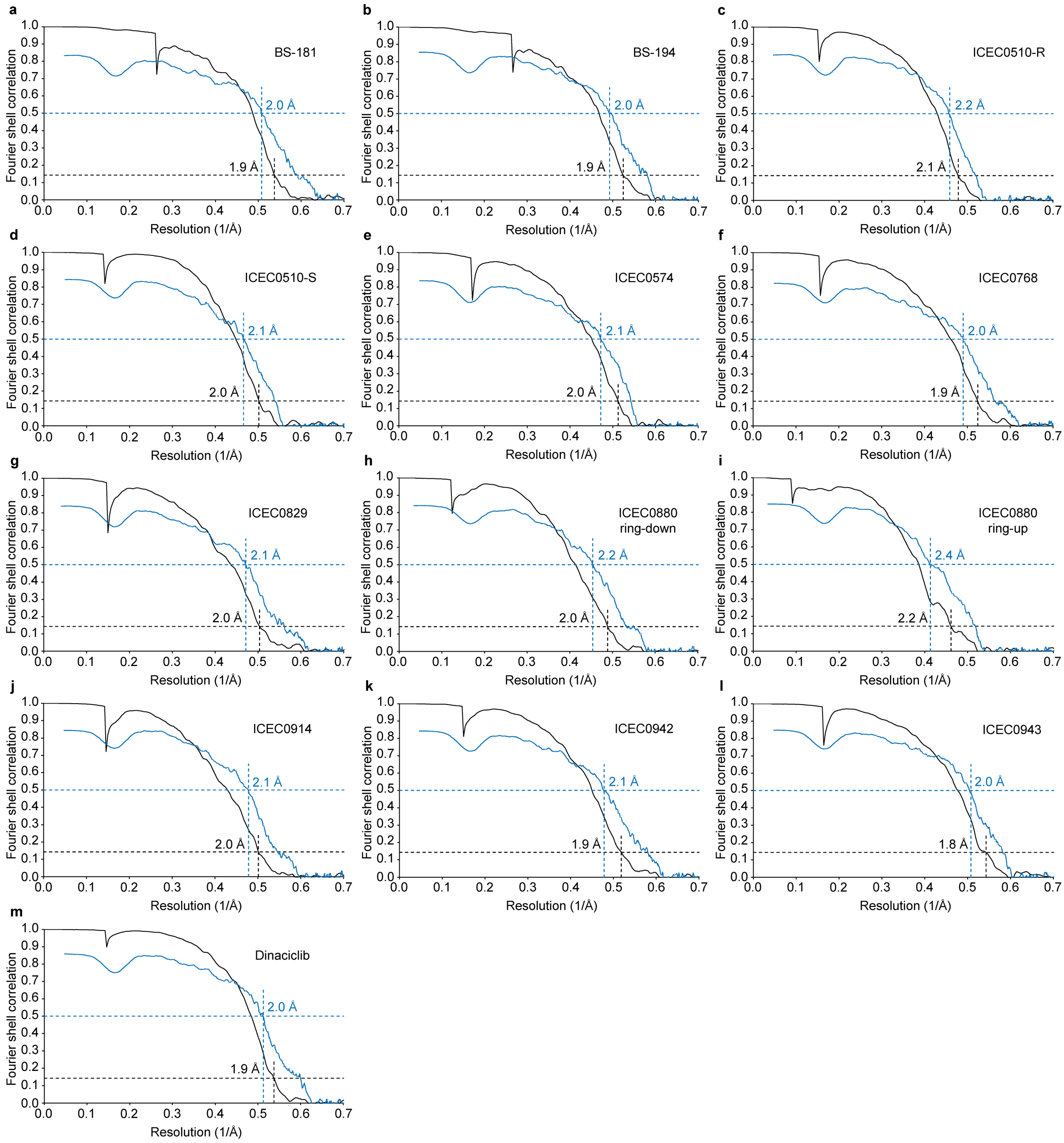

Supplementary Figure 7 | FSC curves for high-resolution inhibitor-bound structures. (a-m) The half-map FSC is shown in black, the model vs. map FSC is shown in blue. Resolution values are estimated from the FSC = 0.143 criterion for half-maps, and the FSC = 0.5 criterion for model vs. map FSCs[1]. Source data are provided as a Source Data file.

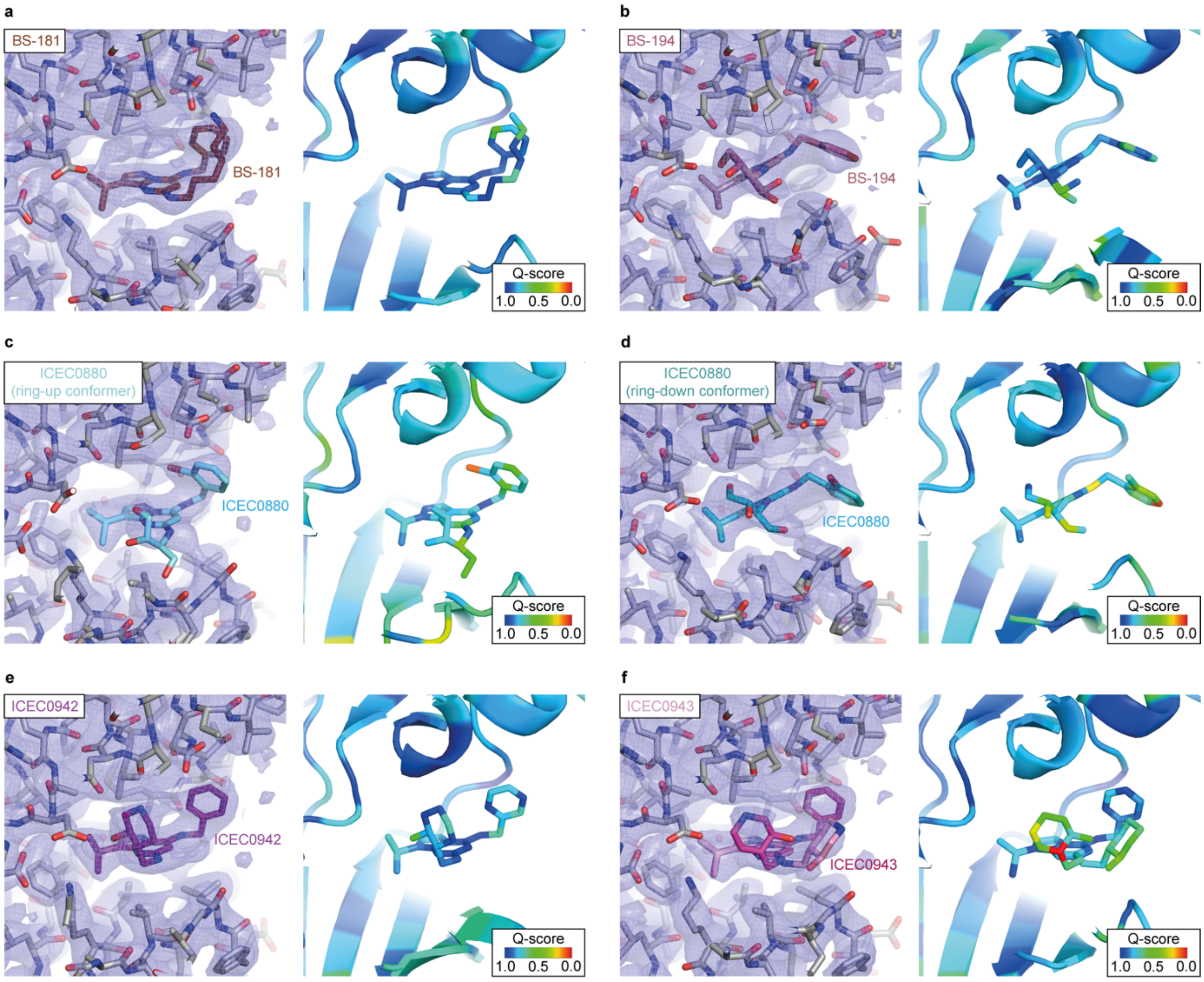

Supplementary Figure 8 | Structures of pyrazolopyrimidine-type inhibitors bound to CDK7 shown with the corresponding cryo-EM maps and evaluated using Q-scores (part 1). Panels show the fitted inhibitors in maps filtered to 3 Å resolution to visualize poorly ordered chemical groups (blue, left-hand panels) and inhibitors colored by Q-scores (red to blue, right-hand panels). The molecular environment and high-resolution map fit are shown in Fig. 6 for these inhibitors. (**a**) BS-181. (**b**) BS-194. (**c**, **d**) ICEC0880 (two different positions correlated with conformational changes in CDK7). (**e**) The clinical inhibitor ICEC0942. (**f**) ICEC0943, the enantiomer of ICEC0942 (two conformers differing in the position of the hydroxypiperidine substituent).

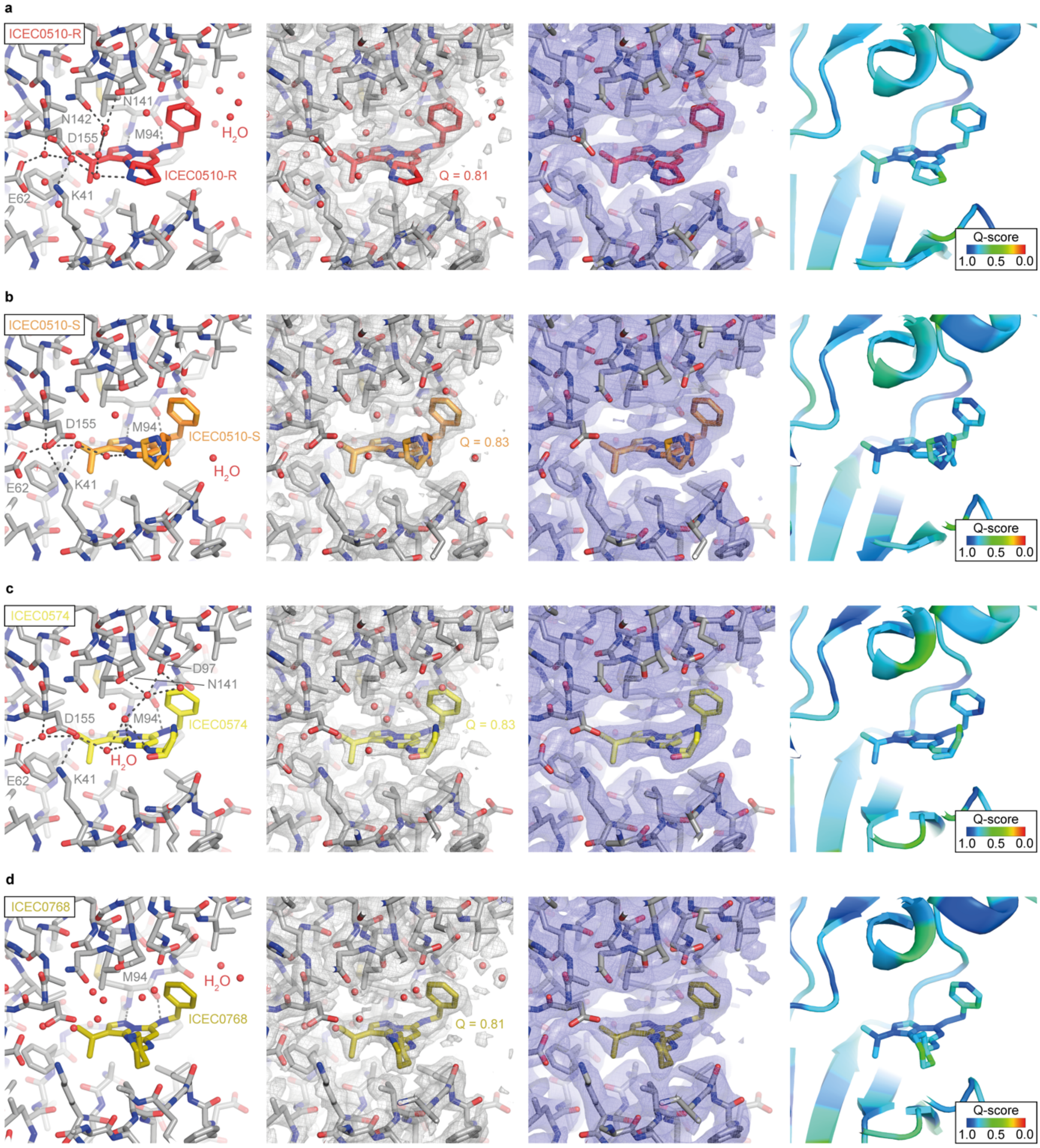

Supplementary Figure 9 | Structures of pyrazolopyrimidine-type inhibitors bound to CDK7 shown with the corresponding cryo-EM maps and evaluated using Q-scores (part 2). From left to right, panels show the inhibitors and their hydrogen bonding interactions when bound to CDK7, fitted inhibitors in the post-processed cryo-EM map (grey; average inhibitor Q-scores are indicated), inhibitors fitted in maps filtered to 3 Å resolution to visualize poorly ordered chemical groups (blue), and inhibitors colored by Q-scores (red to blue, right-hand panels). (**a**) ICEC510-R. (**b**) ICEC510-S (two conformers). (**c**) ICEC0574. (**d**) ICEC0768.

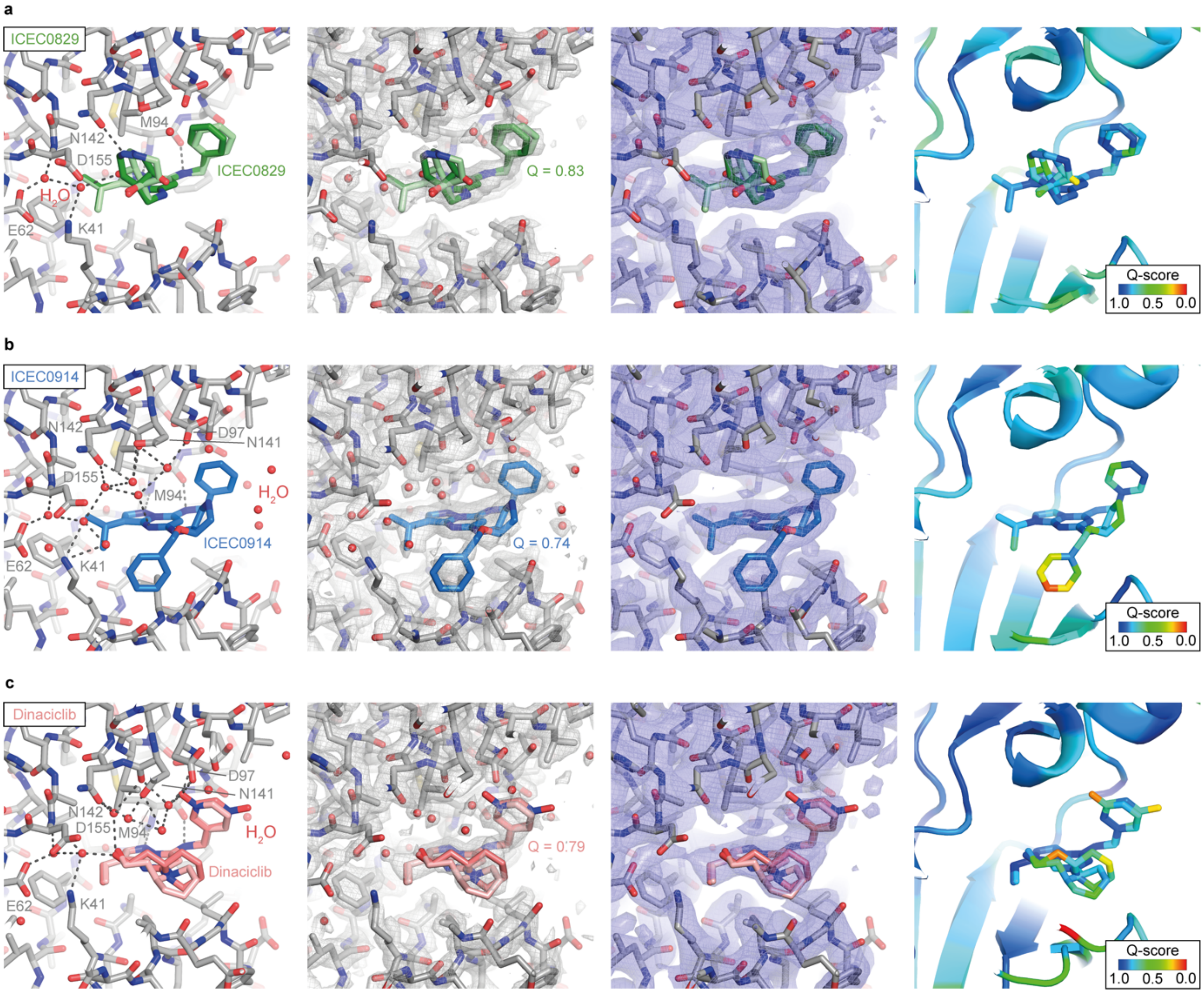

Supplementary Figure 10 | Structures of pyrazolopyrimidine-type inhibitors bound to CDK7 shown with the corresponding cryo-EM maps and evaluated using Q-scores (part 3). From left to right, panels show the inhibitors and their hydrogen bonding interactions when bound to CDK7, fitted inhibitors in the post-processed cryo-EM map (grey; average inhibitor Q-scores are indicated), inhibitors fitted in maps filtered to 3 Å resolution to visualize poorly ordered chemical groups (blue), and inhibitors colored by Q-scores (red to blue, right-hand panels). (**a**) ICEC0829 (two enantiomers). (**b**) ICEC0914. (**c**) Dinaciclib (two conformers because the nitroxide position could not be assigned unambiguously).

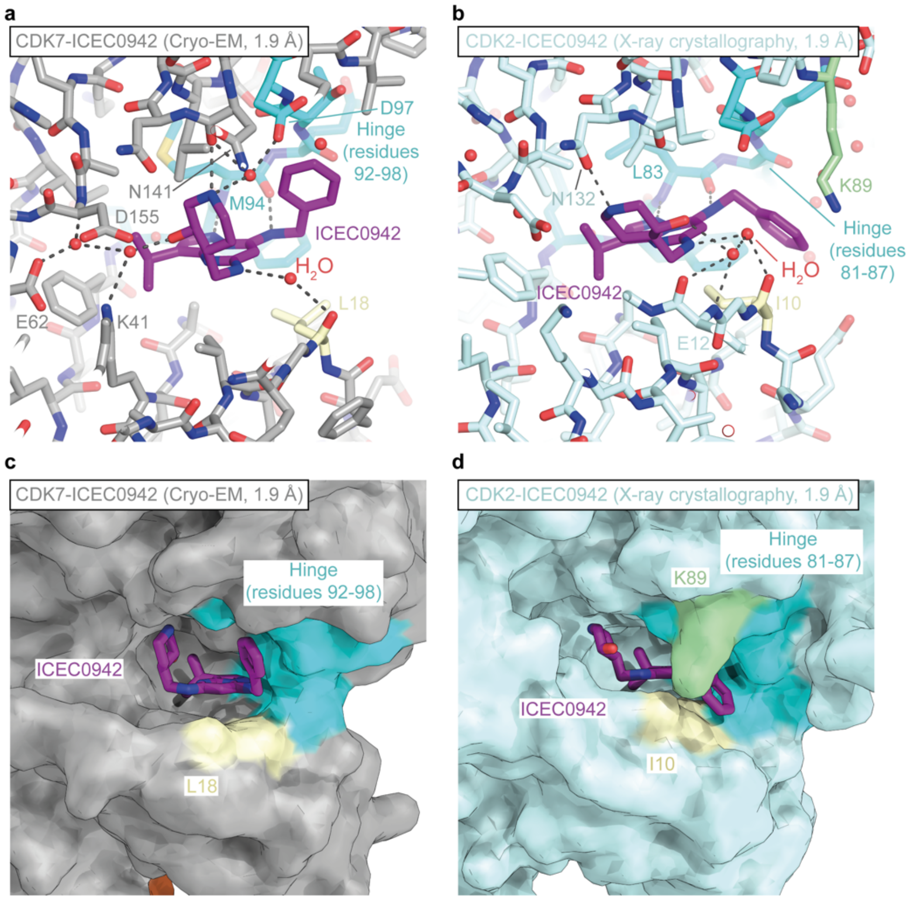

Supplementary Figure 11 | Comparison of ICEC0942-CDK7 and ICEC0942-CDK2 structures and implications for mechanism of selectivity. (**a**) Structure of ICEC0942 (purple) bound to CDK7 (grey; hinge region teal, L18 yellow) in the human CAK. (**b**) The interactions of ICEC0942 in the CDK2 complex (PDB ID 5JQ5, ref. [5]; CDK2 cyan, hinge region teal, I10 yellow, K89 green). (**c**, **d**) Visualization of the active site pockets of CDK7 (c) and CDK2 (d) with bound ICEC0942. A detailed analysis of inhibitor interactions is provided in the main text.

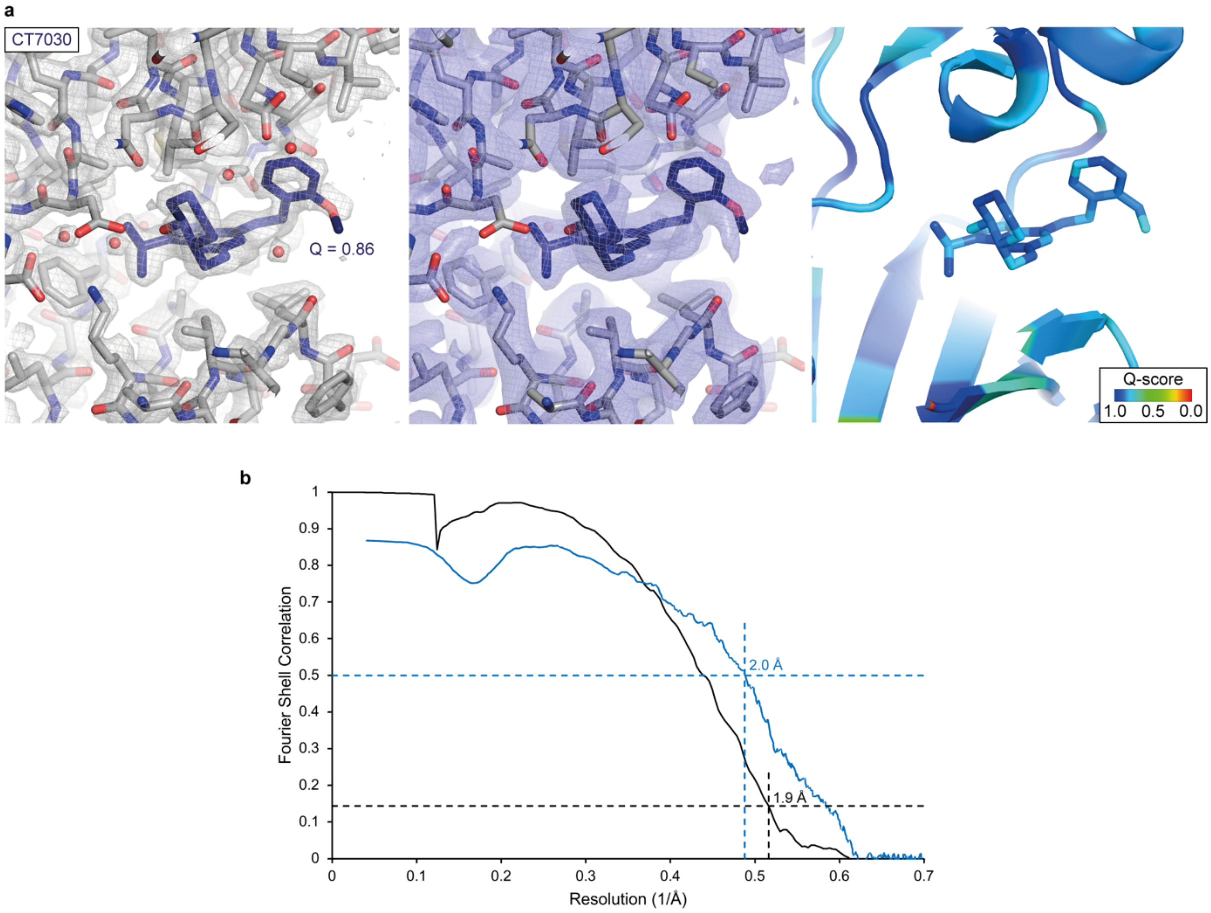

Supplementary Figure 12 | Structures of CT7030 bound to CDK7 shown with the corresponding cryo-EM maps and FSC curve for this reconstruction. (**a**) Details of the CAK-CT7030 structure. Panels show the fitted inhibitor in the post-processed cryo-EM map (grey, left side panel), inhibitor fitted in the map filtered to 3 Å resolution to visualize poorly ordered chemical groups (blue, middle panel), and inhibitor colored by Q-scores (red to blue, right-hand panel). (**b**) FSC curve for the CT7030 cryo-EM structure. The half-map FSC is shown in black, the model vs. map FSC is shown in blue. Resolution values are estimated from the FSC = 0.143 criterion for half-maps, and the FSC = 0.5 criterion for model vs. map FSCs[1]. Source data are provided as a Source Data file.

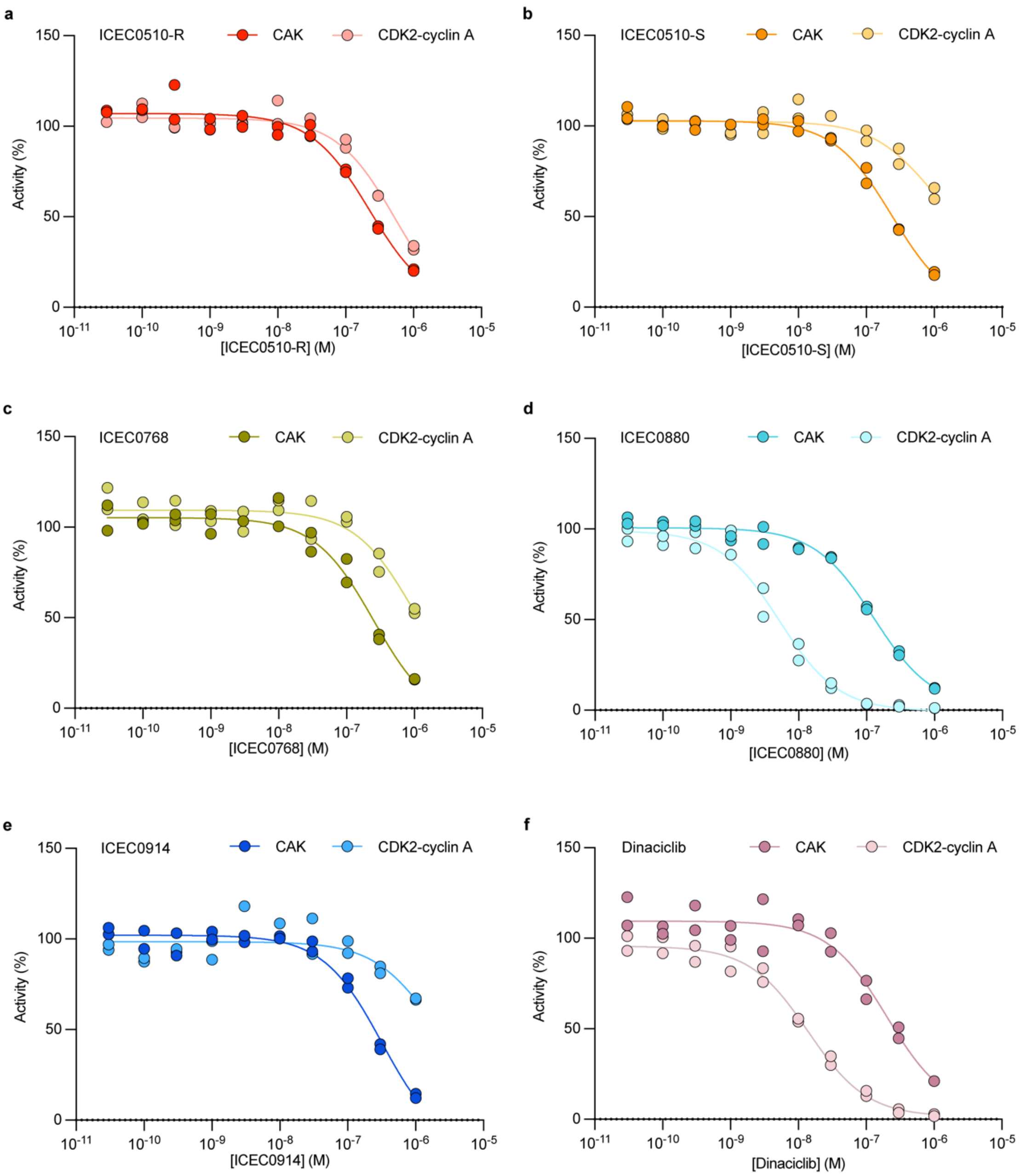

Supplementary Figure 13 | In-vitro enzyme inhibition assay data. Plots of the data underlying the values reported in Supplementary Table 2 (n=2, with measurements taken in distinct wells). (**a**) ICEC0510-R. (**b**) ICEC0510-S. (**c**) ICEC0768. (**d**) ICEC0880. (**e**) ICEC0914. (**f**) Dinaciclib. Source data are provided as a Source Data file.

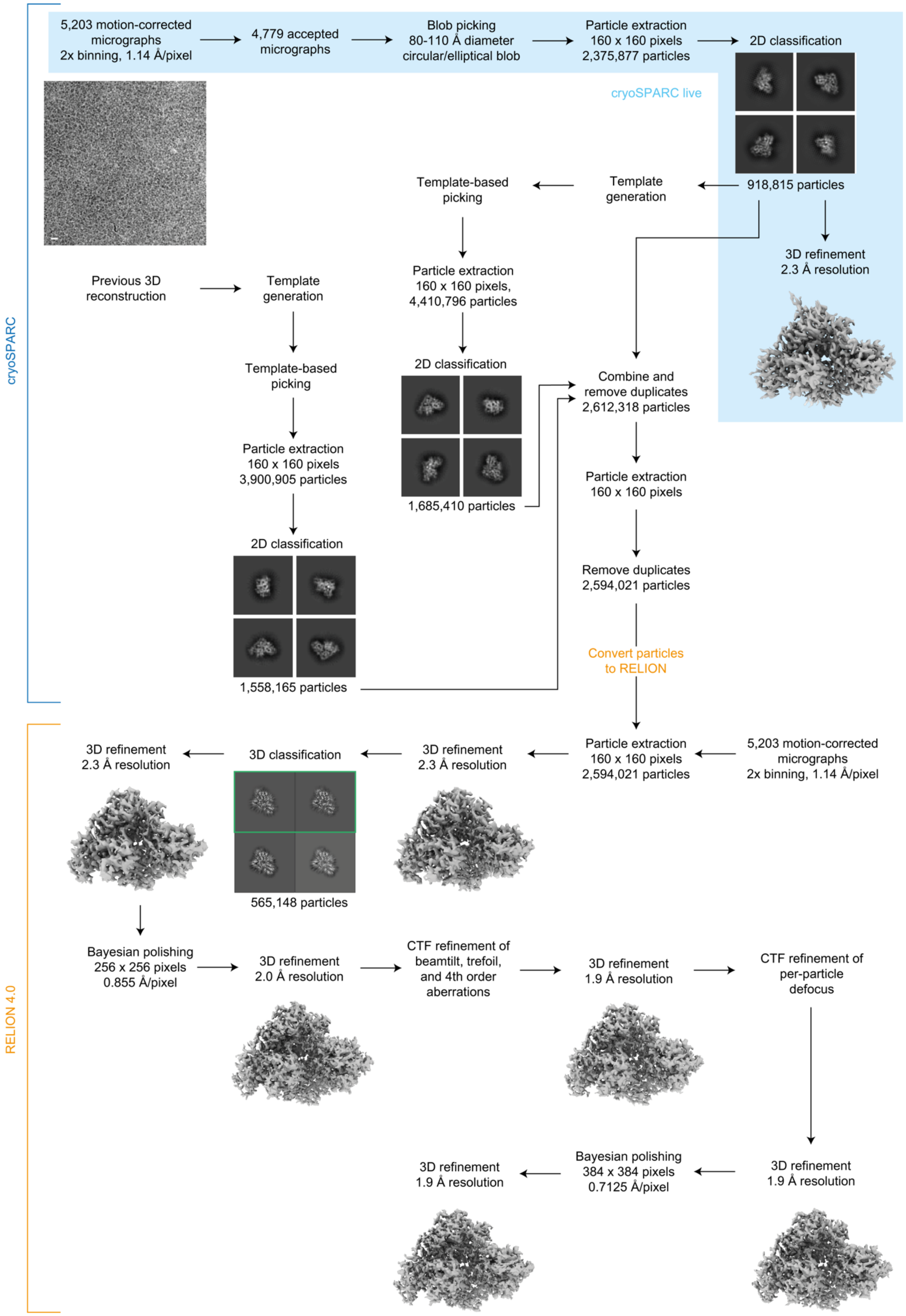

cryoSPARC
5,203 motion-corrected micrographs 2x binning, 1.14 Å/pixel
4,779 accepted micrographs
Blob picking 80-110 Å diameter circular/elliptical blob
Particle extraction 160 x 160 pixels 2,375,877 particles
2D classification
cryoSPARC live
918,815 particles
Template generation
Template-based picking
3D refinement 2.3 Å resolution
Previous 3D reconstruction
Template generation
Particle extraction 160 x 160 pixels, 4,410,796 particles
Template-based picking
2D classification
Combine and remove duplicates 2,612,318 particles
Particle extraction 160 x 160 pixels 3,900,905 particles
1,685,410 particles
Particle extraction 160 x 160 pixels
2D classification
Remove duplicates 2,594,021 particles
Convert particles to RELION
1,558,165 particles
RELION 4.0
3D refinement 2.3 Å resolution
3D classification
3D refinement 2.3 Å resolution
Particle extraction 160 x 160 pixels 2,594,021 particles
5,203 motion-corrected micrographs 2x binning, 1.14 Å/pixel
565,148 particles
Bayesian polishing 256 x 256 pixels 0.855 Å/pixel
3D refinement 2.0 Å resolution
CTF refinement of beamtilt, trefoil, and 4th order aberrations
3D refinement 1.9 Å resolution
CTF refinement of per-particle defocus
3D refinement 1.9 Å resolution
Bayesian polishing 384 x 384 pixels 0.7125 Å/pixel
3D refinement 1.9 Å resolution

Supplementary Figure 14 | Data processing strategy. Processing stages using cryoSPARC and RELION are indicated. Steps performed in cryoSPARC live are shaded in blue. A representative micrograph is shown (scale bar: 100 Å); the contrast of the micrograph was enhanced, and a low-pass filter was applied to facilitate visualization of the particles.

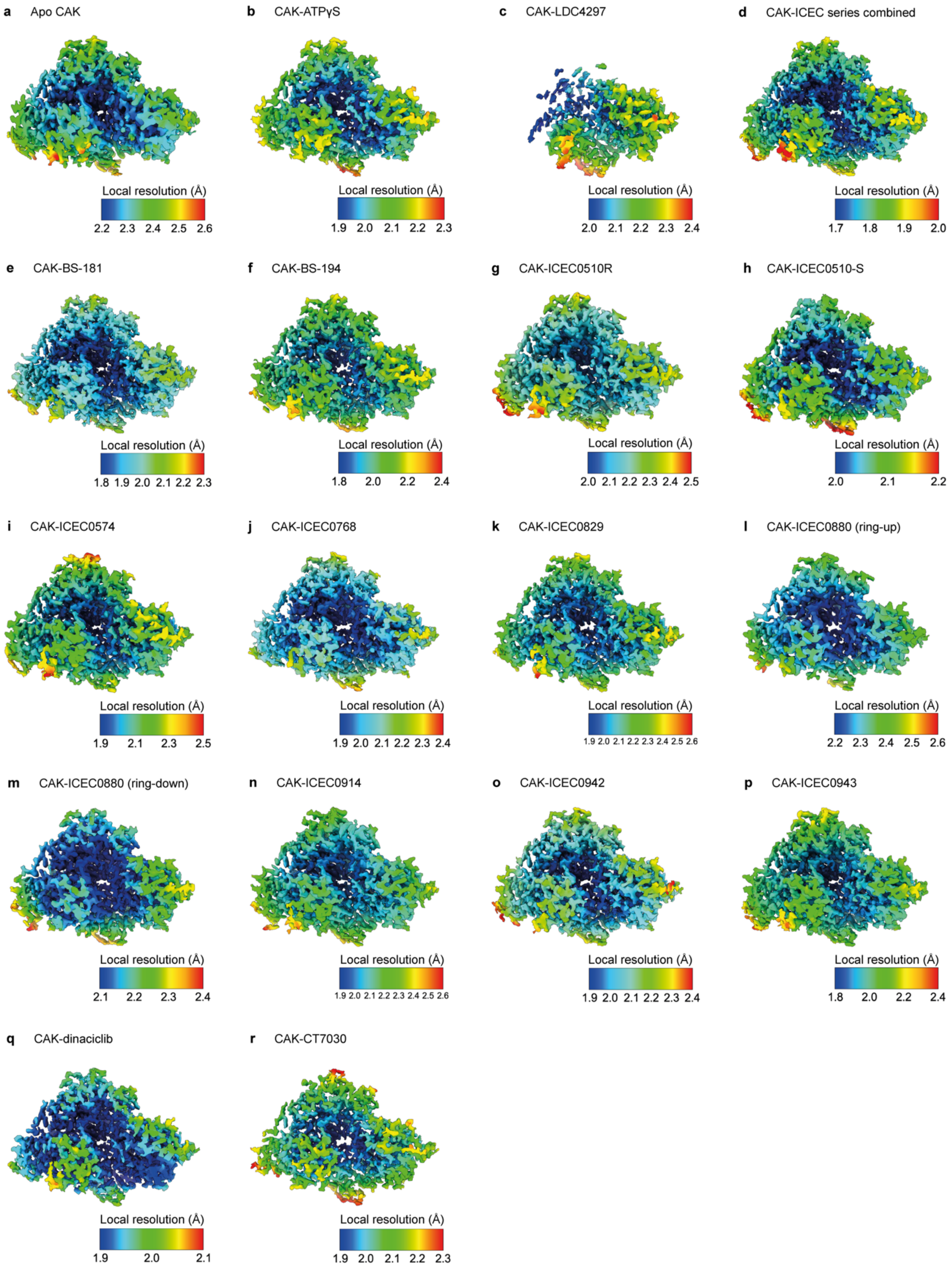

Supplementary Figure 15 | Local resolution estimates. (**a**-**r**) Local resolution estimates for all high-resolution reconstructions obtained during this study except CAK-THZ1. Local resolutions were computed using RELION. The corresponding depiction for CAK-THZ1 is provided in Fig. 1c.

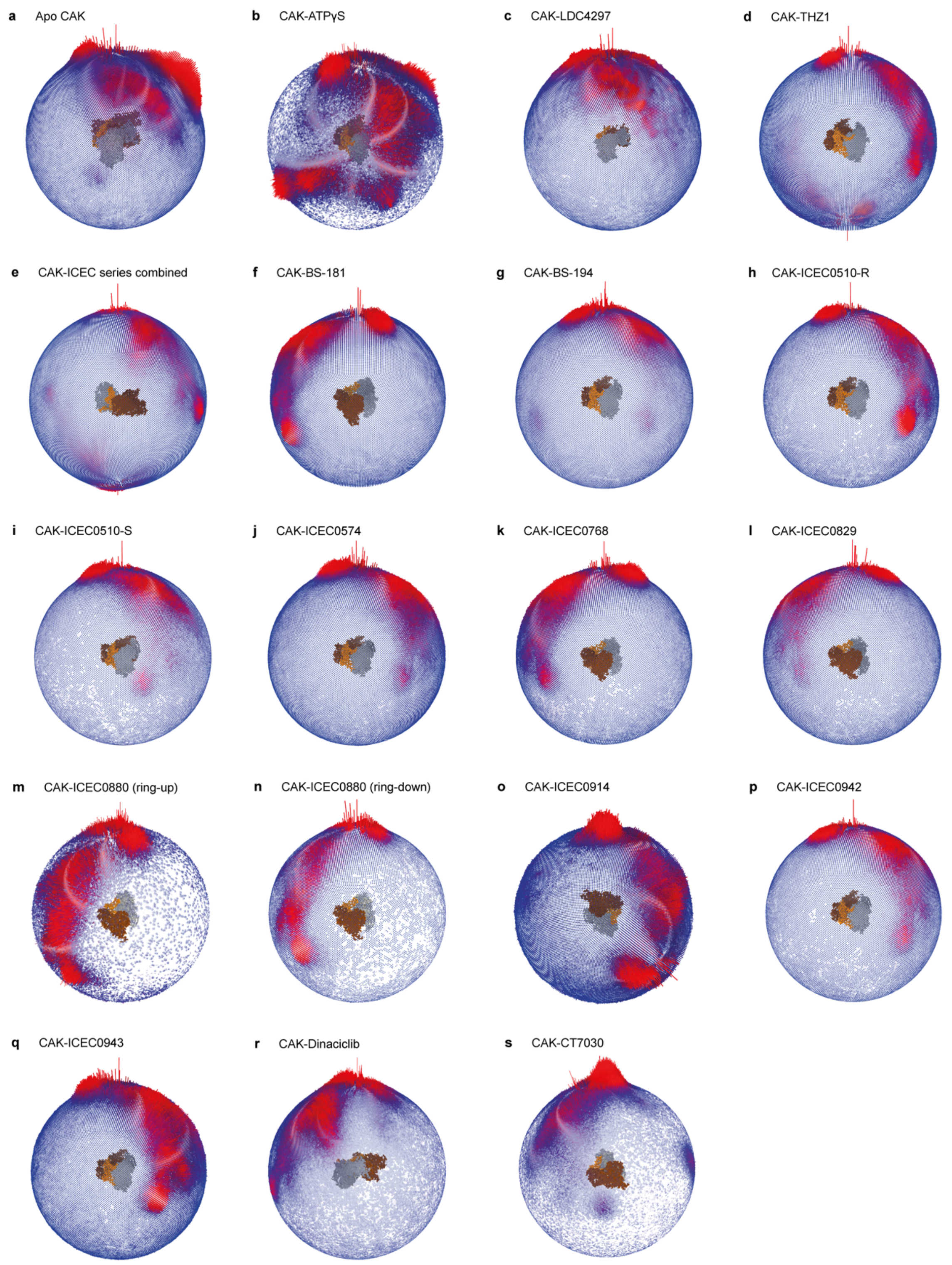

Supplementary Figure 16 | Orientation distributions. (a-s) Orientation distribution plots for all high-resolution reconstructions obtained during this study.

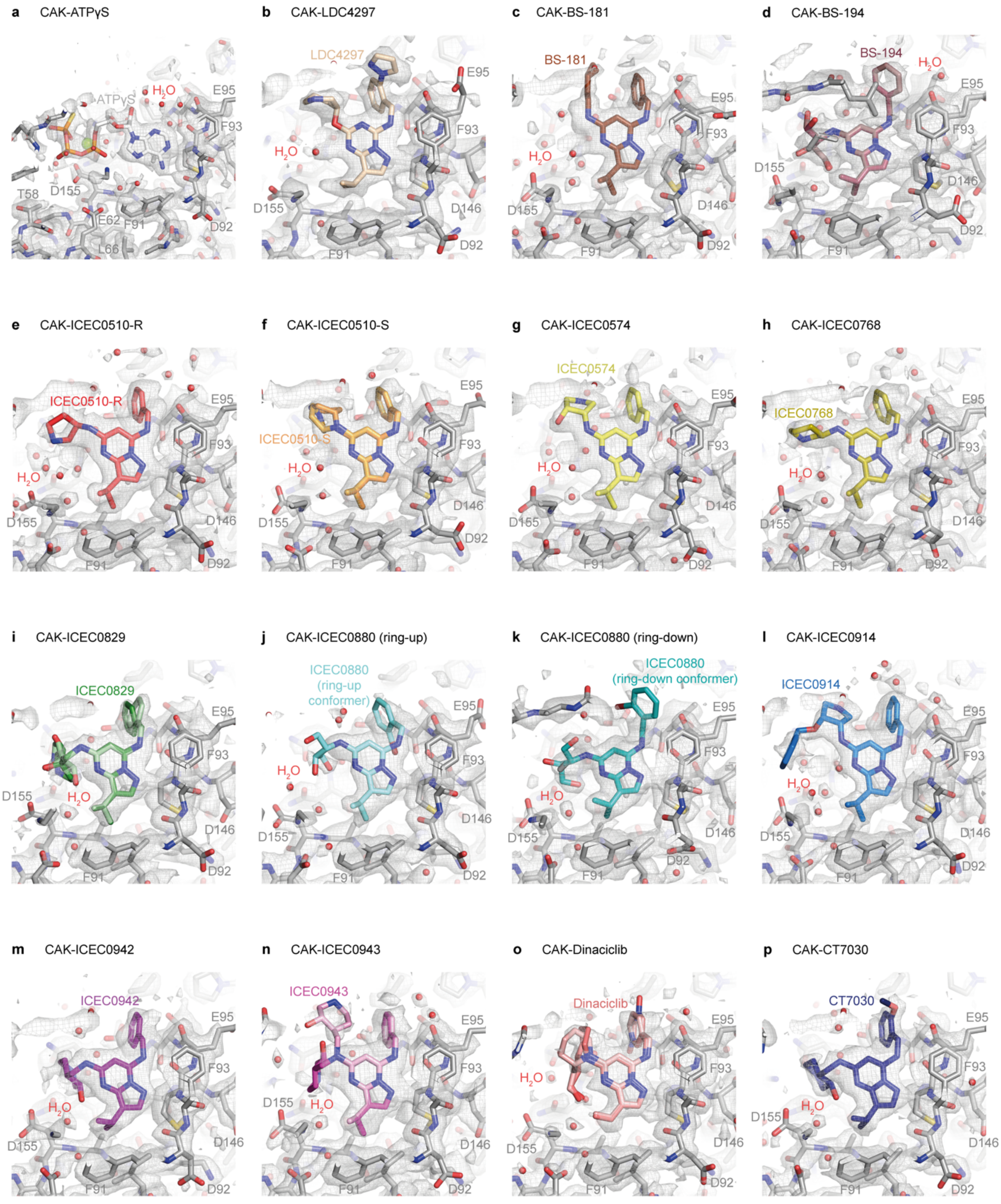

Supplementary Figure 17 | Additional ligand views. (**a**-**p**) A second view of all high-resolution ligand densities shown in Fig. 6 and Supplementary Figs 1, 2, 9, 10, and 12.

Supplementary Table 1 | Krios G4 datasets collected during this study.

Ligand	Grid	Micrographs collected	Micrographs accepted	Particles refined (live)	Resolution cryoSPARC (Å) [a]	Particles refined (final)	Resolution RELION (Å)
apo	VC15-1	5,805	5,036	1,402,951	2.3	486,457	2.3
ATPγS	VC16-3	5,781	5,238	830,780	2.3	126,177	1.9
THZ1	BG29-1	6,029	5,015	932,552	2.3	555,117	1.9
	BG29-2	6,542	5,553	850,654	2.3		
LDC4297	BG30-3	9,553	6,426	835,340	2.5	273,097	2.1
BS-181	VC8-1	5,203	4,779	918,185	2.3	565,148	1.9
BS-194	VC9-4	6,351	5,725	1,041,685	2.3	688,511	1.9
ICEC0510-R	VC6-4	6,309	5,130	981,580	2.5	268,647	2.1
ICEC0510-S	VC7-3	5,081	3,564	916,457	2.3	233,209	2.0
ICEC0574	VC11-4	5,132	3,400	770,136	2.4	410,939	2.0
ICEC0768	VC2-4	5,583	4,038	803,921	2.3	334,554	1.9
ICEC0829	VC12-1	5,502	3,925	688,486	2.4	253,285	2.0
	VC12-3	5,079	1,990	390,255	2.5	171,002	2.0
	VC12-3 [b]	5,167	2,457	211,647	2.8	165,849	2.4
ICEC0880	VC5-1	5,426	4,173	621,371	2.4	158,290	2.0
	State 1 [c]					112,809	2.0
	State 2 [c]					45,481	2.2
ICEC0914	VC3-1	5,202	3,823	709,706	2.3	237,743	2.0
ICEC0942	VC13-3	5,231	4,423	1,010,580	2.4	302,014	1.9
	VC14-1	5,367	3,885	790,254	2.3	269,962	1.9
ICEC0943	VC1-1	5,729	4,649	959,204	2.3	294,085	1.8
Dinaciclib	VC4-3	5,210	4,561	776,307	2.3	294,740	1.9
CT7030	BG51-1	10,015	5,692	895,333	2.3	459,893	1.9

[a] limited to 2.28 Å (Nyquist frequency) in pre-processing due to 2x binning of input movies
[b] energy filter slit retracted
[c] state 1: ring-down conformation; state 2: ring-up conformation

Supplementary Table 2 | Enzyme inhibition properties of pyrazolopyrimidine-type used for structure determination.

Enzyme inhibition properties of selected compounds. Half-maximal inhibitory concentrations (IC_{50}) of pyrazolopyrimidine inhibitors used in this study determined by *in-vitro* kinase assays are summarized. Enzyme inhibition raw data for values reported in this work are provided in Supplementary Figure 13.

Compound	R1	R2	R3	CDK7 (IC_{50} nM)	CDK2 (IC_{50} nM)	Selectivity	Reference
BS181	-$(CH_2)_6NH_2$	Bn	CH_3	21	880	42	Ref. [6]
BS194	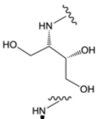	Bn	CH_3	250	3	0.01	Ref. [7]
ICEC0510-R	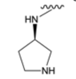	Bn	CH_3	220	540	2	This work
ICEC0510-S	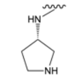	Bn	CH_3	240	880	4	This work
ICEC0574	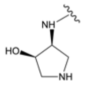	Bn	CH_3	27	1290	48	Ref. [8]
ICEC0768	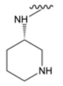	Bn	CH_3	250	970	4	This work
ICEC0829	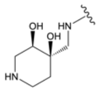	Bn	CH_3	18	2030	113	Ref. [8]
ICEC0880	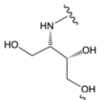	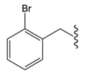	CH_3	130	5	0.04	This work
ICEC0914	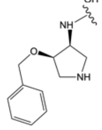	Bn	CH_3	320	>1000	-	This work
ICEC0942	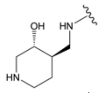	Bn	CH_3	41	578	14	Ref. [5]
ICEC0943	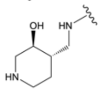	Bn	CH_3	925	>1000	-	Ref. [5]
Dinaciclib	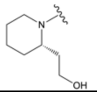	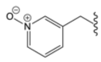	H	200	14	0.07	This work

Abbreviations: Bn - benzyl group, $-CH_2-C_6H_5$

Supplementary Table 3 | Glacios 2 1-hour screening datasets collected during this study. Grids selected for the next step in the data collection pipeline are marked in bold.

Ligand	Grid	Micrographs collected	Micrographs accepted	Particles refined (live)	Resolution cryoSPARC live (Å)
BS-181	VC8-1	452	306	32,405	3.7
	**VC8-2**	460	335	57,762	3.2
BS-194	VC9-3	488	413	40,610	3.6
	**VC9-4**	502	421	61,014	3.6
ICEC0510-R	VC6-3	422	318	42,637	3.4
	**VC6-4**	511	262	31,111	3.6
ICEC0510-S	**VC7-3**	452	344	45,237	3.4
	VC7-4	466	299	40,464	3.4
ICEC0574	VC11-3	454	278	35,753	4.1
	**VC11-4**	448	222	17,099	3.9
ICEC0768	**VC2-1**	407	276	22,322	4.3
	VC2-2	457	318	27,998	4.9
ICEC0829	**VC12-3**	426	240	26,990	3.5
	VC12-4	423	193	30,776	3.7
ICEC0880	**VC5-1**	600	391	51,397	3.8
	VC5-2	600	482	65,836	3.8
ICEC0914	**VC3-1**	458	358	33,750	4.5
	VC3-2	486	163	17,928	4.1
ICEC0942	**VC13-1**	634	362	35,220	3.6
	VC13-2	600	373	24,151	4.1
	**VC14-1**	464	345	67,854	3.3
	VC14-2	450	302	31,000	3.5
ICEC0943	**VC1-1**	470	310	31,034	3.4
	VC1-2	452	321	41,690	3.5
Dinaciclib	**VC4-1**	466	344	57,102	3.5
	VC4-2	453	317	25,137	4.0

Supplementary Table 4 | Glacios 2 4-hour screening datasets collected during this study.

Ligand	Grid	Micrographs collected	Micrographs accepted	Particles refined (live)	Resolution cryoSPARC live (Å)	Particles refined (final)	Resolution RELION (Å)
BS-181	VC8-2	1,686	1,281	201,719	3.0	93,194	3.1
BS-194	VC9-4	1,659	1,326	282,212	3.1	55,890	3.2
ICEC0510-R	VC6-4	1,678	1,106	176,583	3.1	59,714	3.1
ICEC0510-S	VC7-3	1,558	1,242	220,033	2.9	166,785	2.9
ICEC0574	VC11-4	1,764	953	156,086	3.0	62,893	3.0
ICEC0768	VC2-1	1,703	1,227	191,294	3.0	149,596	3.0
ICEC0829	VC12-3	1,594	1,110	144,402	3.0	60,827	3.0
ICEC0880	VC5-1 [a]	10,576	7,907	1,232,300	3.3	226,221	2.3
ICEC0914	VC3-1	1,632	1,217	145,713	3.0	47,422	3.0
ICEC0942	VC13-1	1,776	1,007	143,912	3.3	62,044	3.2
	VC14-1	1,658	1,285	246,409	3.1	134,727	3.2
ICEC0943	VC1-1	1,202	783	95,031	2.9	60,500	3.0
Dinaciclib	VC4-1	1,637	1,194	160,000	3.2	128,172	3.1

[a] full-size dataset for performance comparison with Krios G4

Supplementary Table 5 | Cryo-EM data collection, 3D reconstruction, and refinement statistics, part 1.

**Ligand**	**apo-CAK**	**ATPγS**	**LDC4297**
Microscope	Krios G4	Krios G4	Krios G4
Stage type	Autoloader	Autoloader	Autoloader
Voltage (kV)	300	300	300
Detector	Falcon 4i	Falcon 4i	Falcon 4i
Acquisition mode	Counting	Counting	Counting
Physical pixel size (Å)	0.57	0.57	0.57
Defocus range (µm)	0.8-1.4	0.7-1.5	0.4-2.0
Electron exposure (e^-/A^2)	70	70	70
**Reconstruction**	**EMD-17523**	**EMD-17511**	**EMD-17536**
Software	RELION 4.0	RELION 4.0	RELION 4.0
Particles used	486,457	126,177	272,469
Box size (pixels)	256 x 256 x 256	384 x 384 x 384	256 x 256 x 256
Final pixel size (Å)	0.855	0.7125	0.855
Accuracy rotations (°)	0.98	0.60	1.07
Accuracy translations (Å)	0.29	0.21	0.31
Map resolution (Å)	2.3	1.9	2.1
Map resolution range (Å)	2.2-2.6	1.9-2.3	2.0-2.5
Sphericity	0.86	0.91	0.88
Map sharpening B-factor ($Å^2$)	-51	-18	-35
**Coordinate refinement**			
Software	N/A	PHENIX	PHENIX-OPLS4
Refinement algorithm	N/A	REAL SPACE	REAL SPACE
Resolution cutoff (Å)	N/A	1.9	2.1
$FSC_{model\text{-}vs\text{-}map}$=0.5 (Å)	N/A	2.0	2.4
**Model**	N/A	**PDB-8P6Y**	**PDB-8P7L**
Number of residues	N/A	791	358
Protein	N/A	630	315
Ligand (inhibitor, Mg^{2+})	N/A	3	1
Water	N/A	158	42
B-factors overall	N/A	16.67	25.55
Protein	N/A	16.63	25.73
Ligand (inhibitor, Mg^{2+})	N/A	29.05	19.27
Water	N/A	14.98	19.84
R.M.S. deviations			
Bond lengths (Å)	N/A	0.002	0.003
Bond angles (°)	N/A	0.521	0.593
**Validation**			
Molprobity score	N/A	1.18	1.80
Molprobity clashscore	N/A	3.92	10.93
Rotamer outliers (%)	N/A	0.36	1.11
C_β deviations (%)	N/A	0.00	0.00
Ramachandran plot			
Favored (%)	N/A	98.87	96.72
Allowed (%)	N/A	0.97	2.95
Outliers (%)	N/A	0.16	0.33

Supplementary Table 6. Cryo-EM data collection, 3D reconstruction, and refinement statistics, part 2.

**Ligand**	**THZ1 (grid BG29-1)**	**THZ1 (grid BG29-2)**	**ICEC series combined**
Microscope	Krios G4	Krios G4	Krios G4
Stage type	Autoloader	Autoloader	Autoloader
Voltage (kV)	300	300	300
Detector	Falcon 4i	Falcon 4i	Falcon 4i
Acquisition mode	Counting	Counting	Counting
Physical pixel size (Å)	0.57	0.57	0.57
Defocus range (µm)	0.4-1.9	0.4-1.8	-
Electron exposure (e^-/A^2)	70	70	70
**Reconstruction**	**EMD-17129**		**EMD-17522**
Software	RELION 4.0		RELION 4.0
Particles used	555,117		2,060,503
Box size (pixels)	384 x 384 x 384		384 x 384 x 384
Final pixel size (Å)	0.7125		0.7125
Accuracy rotations (°)	0.67		0.64
Accuracy translations (Å)	0.22		0.22
Map resolution (Å)	1.9		1.7
Map resolution range (Å)	1.8-2.3		1.7-2.0
Sphericity	0.90		0.88
Map sharpening B-factor (Å^2)	-28		-27
**Coordinate refinement**			
Software	PHENIX		SERVALCAT/ REFMAC
Refinement algorithm	REAL SPACE		Reciprocal space
Resolution cutoff (Å)	1.9		1.7
$FSC_{model-vs-map}$=0.5 (Å)	1.9		1.8
**Model**	**PDB-8ORM**		**PDB-8P79**
Number of residues	844		811
Protein	632		630
Ligand (inhibitor, Mg^{2+})	2		1
Water	210		180
B-factors overall	10.68		33.58
Protein	10.56		33.69
Ligand (inhibitor, Mg^{2+})	18.73		15.11
Water	11.85		30.60
R.M.S. deviations			
Bond lengths (Å)	0.003		0.014
Bond angles (°)	0.536		1.719
**Validation**			
Molprobity score	1.22		1.23
Molprobity clashscore	4.40		3.84
Rotamer outliers (%)	0.90		0.90
C_β deviations (%)	0.00		0.00
Ramachandran plot			
Favored (%)	98.23		97.73
Allowed (%)	1.61		2.10
Outliers (%)	0.16		0.16

Supplementary Table 7 | Cryo-EM data collection, 3D reconstruction, and refinement statistics, part 3.

**Ligand**	**BS-181**	**BS-194**	**ICEC0510-R**
Microscope	Krios G4	Krios G4	Krios G4
Stage type	Autoloader	Autoloader	Autoloader
Voltage (kV)	300	300	300
Detector	Falcon 4i	Falcon 4i	Falcon 4i
Acquisition mode	Counting	Counting	Counting
Physical pixel size (Å)	0.57	0.57	0.57
Defocus range (µm)	0.4-1.5	0.4-1.8	0.4-1.6
Electron exposure (e^-/A^2)	70	70	70
**Reconstruction**	**EMD-17509**	**EMD-17510**	**EMD-17512**
Software	RELION 4.0	RELION 4.0	RELION 4.0
Particles used	565,148	688,511	268,647
Box size (pixels)	384 x 384 x 384	384 x 384 x 384	384 x 384 x 384
Final pixel size (Å)	0.7125	0.7125	0.7125
Accuracy rotations (°)	0.68	0.84	0.71
Accuracy translations (Å)	0.23	0.25	0.23
Map resolution (Å)	1.9	1.9	2.1
Map resolution range	1.8-2.3	1.8-2.4	2.0-2.5
Sphericity	0.84	0.86	0.86
Map sharpening B-factor ($Å^2$)	-27	-26	-28
**Coordinate refinement**			
Software	PHENIX	PHENIX	PHENIX
Refinement algorithm	REAL SPACE	REAL SPACE	REAL SPACE
Resolution cutoff (Å)	1.9	1.9	2.1
$FSC_{model\text{-}vs\text{-}map}$=0.5 (Å)	2.0	2.0	2.2
**Model**	**PDB-8P6W**	**PDB-8P6X**	**PDB-8P6Z**
Number of residues	771	777	759
Protein	630	631	630
Ligand (inhibitor, Mg^{2+})	2	2	2
Water	139	144	127
B-factors overall	7.69	22.92	22.28
Protein	7.74	23.12	22.28
Ligand (inhibitor, Mg^{2+})	5.40	14.98	19.97
Water	6.19	19.09	18.98
R.M.S. deviations			
Bond lengths (Å)	0.004	0.003	0.003
Bond angles (°)	0.640	0.561	0.577
**Validation**			
Molprobity score	1.42	1.53	1.37
Molprobity clashscore	7.16	8.20	6.67
Rotamer outliers (%)	0.90	0.72	0.72
C_β deviations (%)	0.00	0.00	0.00
Ramachandran plot			
Favored (%)	97.90	97.58	98.38
Allowed (%)	1.94	2.26	1.46
Outliers (%)	0.16	0.16	0.16

Supplementary Table 8 | Cryo-EM data collection, 3D reconstruction, and refinement statistics, part 4.

**Ligand**	**ICEC0510-S**	**ICEC0574**	**ICEC0768**
Microscope	Krios G4	Krios G4	Krios G4
Stage type	Autoloader	Autoloader	Autoloader
Voltage (kV)	300	300	300
Detector	Falcon 4i	Falcon 4i	Falcon 4i
Acquisition mode	Counting	Counting	Counting
Physical pixel size (Å)	0.57	0.57	0.57
Defocus range (µm)	0.4-1.9	0.4-1.8	0.5-1.7
Electron exposure (e^-/A^2)	70	70	70
**Reconstruction**	**EMD-17513**	**EMD-17514**	**EMD-17515**
Software	RELION 4.0	RELION 4.0	RELION 4.0
Particles used	233,209	410,939	334,554
Box size (pixels)	384 x 384 x 384	384 x 384 x 384	384 x 384 x 384
Final pixel size (Å)	0.7125	0.7125	0.7125
Accuracy rotations (°)	0.67	0.75	0.68
Accuracy translations (Å)	0.22	0.25	0.23
Map resolution (Å)	2.0	2.0	1.9
Map resolution range	2.0-2.2	1.9-2.7	1.9-2.7
Sphericity	0.92	0.84	0.84
Map sharpening B-factor ($Å^2$)	-21	-25	-24
**Coordinate refinement**			
Software	PHENIX	PHENIX	PHENIX
Refinement algorithm	REAL SPACE	REAL SPACE	REAL SPACE
Resolution cutoff (Å)	2.0	2.0	1.9
$FSC_{model-vs-map}$=0.5 (Å)	2.1	2.1	2.0
**Model**	**PDB-8P70**	**PDB-8P71**	**PDB-8P72**
Number of residues	786	746	756
Protein	630	630	630
Ligand (inhibitor, Mg^{2+})	2	2	2
Water	154	114	124
B-factors overall	28.33	20.78	16.54
Protein	28.58	20.92	16.61
Ligand (inhibitor, Mg^{2+})	17.60	13.68	15.73
Water	24.12	16.37	13.73
R.M.S. deviations			
Bond lengths (Å)	0.003	0.003	0.003
Bond angles (°)	0.548	0.520	0.587
**Validation**			
Molprobity score	1.44	1.40	1.38
Molprobity clashscore	8.02	7.36	6.96
Rotamer outliers (%)	0.90	0.72	0.54
C_β deviations (%)	0.00	0.00	0.00
Ramachandran plot			
Favored (%)	98.54	98.22	98.06
Allowed (%)	1.29	1.62	1.78
Outliers (%)	0.16	0.16	0.16

Supplementary Table 9 | Cryo-EM data collection, 3D reconstruction, and refinement statistics, part 5.

Ligand	ICEC0829	ICEC0880 (Ring-down)	ICEC0880 (Ring-up)
Microscope	Krios G4	Krios G4	
Stage type	Autoloader	Autoloader	
Voltage (kV)	300	300	
Detector	Falcon 4i	Falcon 4i	
Acquisition mode	Counting	Counting	
Physical pixel size (Å)	0.57	0.57	
Defocus range (μm)	0.4-1.9	0.4-1.7	
Electron exposure (e^-/A^2)	70	70	
**Reconstruction**	**EMD-17516**	**EMD-17518**	**EMD-17517**
Software	RELION 4.0	RELION 4.0	RELION 4.0
Particles used	253,285	112,809	45,481
Box size (pixels)	384 x 384 x 384	384 x 384 x 384	384 x 384 x 384
Final pixel size (Å)	0.7125	0.7125	0.7125
Accuracy rotations (°)	0.73	0.68	0.62
Accuracy translations (Å)	0.24	0.22	0.22
Map resolution (Å)	2.0	2.0	2.2
Map resolution range	1.9-2.6	2.0-2.7	2.1-3.6
Sphericity	0.82	0.81	0.82
Map sharpening B-factor ($Å^2$)	-23	-21	-13
**Coordinate refinement**			
Software	PHENIX	PHENIX	PHENIX
Refinement algorithm	REAL SPACE	REAL SPACE	REAL SPACE
Resolution cutoff (Å)	2.0	2.0	2.2
$FSC_{model-vs-map}$=0.5 (Å)	2.1	2.2	2.4
**Model**	**PDB-8P73**	**PDB-8P75**	**PDB-8P74**
Number of residues	696	708	672
Protein	626	630	626
Ligand (inhibitor, Mg^{2+})	3	2	2
Water	67	76	44
B-factors overall	22.03	23.47	28.40
Protein	22.20	23.48	28.38
Ligand (inhibitor, Mg^{2+})	16.54	33.32	40.74
Water	14.46	18.85	21.49
R.M.S. deviations			
Bond lengths (Å)	0.004	0.003	0.003
Bond angles (°)	0.623	0.550	0.580
**Validation**			
Molprobity score	1.60	1.42	1.49
Molprobity clashscore	8.86	7.65	9.19
Rotamer outliers (%)	1.45	0.90	0.91
C_β deviations (%)	0.00	0.00	0.00
Ramachandran plot			
Favored (%)	98.37	98.54	98.37
Allowed (%)	1.47	1.29	1.47
Outliers (%)	0.16	0.16	0.16

Supplementary Table 10 | Cryo-EM data collection, 3D reconstruction, and refinement statistics, part 6.

**Ligand**	**ICEC0914**	**ICEC0942**	**ICEC0943**
Microscope	Krios G4	Krios G4	Krios G4
Stage type	Autoloader	Autoloader	Autoloader
Voltage (kV)	300	300	300
Detector	Falcon 4i	Falcon 4i	Falcon 4i
Acquisition mode	Counting	Counting	Counting
Physical pixel size (Å)	0.57	0.57	0.57
Defocus range (µm)	0.4-2.0	0.4-1.9	0.4-1.7
Electron exposure (e^-/A^2)	70	70	70
**Reconstruction**	**EMD-17519**	**EMD-17508**	**EMD-17520**
Software	RELION 4.0	RELION 4.0	RELION 4.0
Particles used	237,743	269,962	294,085
Box size (pixels)	384 x 384 x 384	384 x 384 x 384	384 x 384 x 384
Final pixel size (Å)	0.7125	0.7125	0.7125
Accuracy rotations (°)	0.73	0.69	0.67
Accuracy translations (Å)	0.24	0.22	0.22
Map resolution (Å)	2.0	1.9	1.8
Map resolution range	1.9-2.5	1.9-2.5	1.8-2.4
Sphericity	0.83	0.84	0.85
Map sharpening B-factor ($Å^2$)	-24	-23	-19
**Coordinate refinement**			
Software	PHENIX	PHENIX, OPLS4	PHENIX
Refinement algorithm	REAL SPACE	REAL SPACE	REAL SPACE
Resolution cutoff (Å)	2.0	1.9	1.8
$FSC_{model-vs-map}$=0.5 (Å)	2.1	2.1	2.0
**Model**	**PDB-8P76**	**PDB-8P6V**	**PDB-8P77**
Number of residues	783	770	769
Protein	630	630	630
Ligand (inhibitor, Mg^{2+})	2	2	2
Water	151	138	137
B-factors overall	18.84	21.44	21.06
Protein	18.84	21.54	21.22
Ligand (inhibitor, Mg^{2+})	19.49	14.37	16.21
Water	18.93	19.06	17.19
R.M.S. deviations			
Bond lengths (Å)	0.003	0.003	0.002
Bond angles (°)	0.572	0.586	0.504
**Validation**			
Molprobity score	1.37	1.42	1.33
Molprobity clashscore	6.76	6.18	5.67
Rotamer outliers (%)	0.90	1.26	1.08
C_β deviations (%)	0.00	0.00	0.00
Ramachandran plot			
Favored (%)	98.54	98.06	98.54
Allowed (%)	1.29	1.78	1.29
Outliers (%)	0.16	0.16	0.16

Supplementary Table 11 | Cryo-EM data collection, 3D reconstruction, and refinement statistics, part 7.

**Ligand**	**Dinaciclib**	**CT7030**
Microscope	Krios G4	Krios G4
Stage type	Autoloader	Autoloader
Voltage (kV)	300	300
Detector	Falcon 4i	Falcon 4i
Acquisition mode	Counting	Counting
Physical pixel size (Å)	0.57	0.57
Defocus range (µm)	0.4-1.8	0.3-1.8
Electron exposure (e^-/A^2)	70	70
**Reconstruction**	**EMD-17521**	**EMD-17754**
Software	RELION 4.0	RELION 4.0
Particles used	294,740	176,991
Box size (pixels)	384 x 384 x 384	384 x 384 x 384
Final pixel size (Å)	0.7125	0.7125
Accuracy rotations (°)	0.60	0.58
Accuracy translations (Å)	0.21	0.19
Map resolution (Å)	1.9	1.9
Map resolution range	1.8-2.1	1.9-2.3
Sphericity	0.91	0.84
Map sharpening B-factor ($Å^2$)	-21	-18
**Coordinate refinement**		
Software	PHENIX	PHENIX
Refinement algorithm	REAL SPACE	REAL SPACE
Resolution cutoff (Å)	1.9	1.9
$FSC_{model\text{-}vs\text{-}map}$=0.5 (Å)	2.0	2.0
**Model**	**PDB-8P78**	**PDB-8PLZ**
Number of residues	802	796
Protein	630	630
Ligand (inhibitor, Mg^{2+})	2	2
Water	170	164
B-factors overall	16.39	19.71
Protein	16.45	19.81
Ligand (inhibitor, Mg^{2+})	16.95	14.72
Water	14.42	17.64
R.M.S. deviations		
Bond lengths (Å)	0.003	0.004
Bond angles (°)	0.596	0.743
**Validation**		
Molprobity score	1.33	1.25
Molprobity clashscore	5.57	4.80
Rotamer outliers (%)	0.72	0.90
C_β deviations (%)	0.00	0.00
Ramachandran plot		
Favored (%)	97.90	98.22
Allowed (%)	1.94	1.62
Outliers (%)	0.16	0.16

Supplementary Table 12 | Results of UV-vis and LC-MS verification of ICEC-series compounds and CT7030.

Ligand	$(M+H)^+$ expected (Da)	$(M+H)^+$ observed (Da)	UV-vis purity (%)	LC-MS purity (%)	Compound purity (%)
BS-181	381.2763	381.2763	>95	>95	>95
BS-194	386.2187	386.2187	>95	>95	>95
ICEC0510-R	351.2291	351.2291	>95	>95	>95
ICEC0510-S	351.2291	351.2295	>95	>95	>95
ICEC0574	367.2243	367.2242	>95	>95	>95
ICEC0768	365.2453	365.2453	94	>95	>95
ICEC0829	411.2506	411.2505	78	83	83
ICEC0880	464.1293	464.1274	95	90	95
ICEC0914	457.2710	457.2716	95	95	95
ICEC0943	395.2555	395.2555	>95	>95	>95
CT7030	425.2660	425.2673	91	87	91

Supplementary Note 1. Comparison of 200 kV Glacios 2 and 300 kV Krios G4 instrument performance

While most high-resolution structures deposited to the Electron Microscopy Data Resource (http://www.emdataresource.org) have been determined from data collected on high-end, 300 kV electron microscopes, more accessible 200 kV microscopes equipped with autoloaders and direct electron detectors are increasingly applied to determination of published structures and atomic models. Their applicability to a variety of specimens, including small soluble complexes [9,10] and small membrane proteins [11], and their ability to achieve better than 2 Å resolution on ideal – i.e. symmetrical, rigid, and often large – targets [12,13] have been demonstrated. However, there is a scarcity of direct comparisons between the results obtained using 200 kV and 300 kV instruments on the exact same specimen.

To address this question and provide a more systematic comparison, we determined the structure of the CDK-activating kinase bound to an inhibitor (see main text) from 4,173 good micrographs (count after removal of poor-quality micrographs) collected on a 300 kV Krios G4 cryo-TEM equipped with a cold-FEG, a Selectris X energy filter, and a Falcon 4i direct electron detector. Using a combination of cryoSPARC [14] and RELION [15] for image processing, we were able to reconstruct a 3D map at 2.0 Å from these data (Fig. 3a, c). Having verified the ability to achieve high resolution from this specimen, we transferred the same grid to a 200 kV Glacios 2 cryo-TEM equipped with a Selectris X energy filter and a Falcon 4i direct electron detector and acquired 7,907 good micrographs (count after removal of poor-quality micrographs). The energy filter and detector were thus identical, while the electron source and the optics were different between these datasets. From this latter setup, we achieved 2.3 Å resolution (Fig. 3a, b). This is an improvement compared to our previous best result at 2.5 Å using a 200 kV Talos Arctica cryo-TEM equipped with a Gatan K3 direct detector [9] and might be attributable to the use of a different camera and energy filtration. However, despite this improvement in the 200 kV-data, the result from the 300 kV-instrument was superior to the map derived from the 200 kV-data, notably from fewer micrographs (4,173 for the Krios G4, 7,907 for the Glacios 2) and within a shorter collection time (10 hours and 22 hours, respectively). To facilitate analysis of these data we computed reconstructions from data subsets and plotted the resulting resolutions (Fig. 3d, e).

We note that this result was obtained with a small (85 kDa) complex that lacks symmetry, a combination that is typically considered challenging [16], and is therefore more representative of high-end biological use cases than experiments with model protein complexes. Results with large or highly symmetric specimens, where alignment accuracy is less limiting due to greater signal, may show different resolution gaps between the two instrument types. We also acknowledge that this comparison is primarily valid for the specific systems used, that the cold-FEG may contribute to the superior results from the 300 kV system, and that the DQE difference between 200 kV and 300 kV data may depend on the direct detector model used.

Supplementary Note 2. Comparison of the impact of energy filtration on the quality of low-defocus cryo-EM data of a small complex

The importance of energy filtration as a means to remove inelastically scattered electrons from the electron beam arriving at the detector, thereby improving the signal-to-noise ratio by reducing electrons that only contribute to the noise but not the signal, is well-established for thick specimens, such as those routinely found in cryo-electron tomography experiments [17]. While many high-resolution single-particle structures obtained from thin specimens employed energy filtration as well [18,19], there are few systematic tests that analyze the effect of energy filtration on the quality of data obtained from thin single-particle specimens of smaller complexes such as the CAK studied in this work. We therefore collected low-defocus (0.5-1.0 μm) data using the standard setup (Krios G4 with cold-FEG, Selectris X energy filter, and Falcon 4i detector) and then proceeded to collect another dataset on the same grid after retraction of the energy filtration slit. We obtained a resolution of 2.0 Å from the energy filtered data and 2.3 Å from the data after retraction of the slit (Fig. 3a). Data were processed as described for all other complexes (see Methods), except that additional instances of 2D classification were run to identify all suitable particles, and that only one round of Bayesian polishing was performed before the final refinement. Refinement of sub-sets showed that the energy filtered data also provide higher resolution for smaller dataset sizes, and that breaking the 2 Å barrier may be difficult without energy filtration for our specimen and data collection parameters (Fig. 3f, g).

We note that our experiment strongly supports a positive effect of energy filtration on the data quality of the 85 kDa human CAK collected at low defocus, but that further experiments are required to investigate if the conclusions drawn from this comparison can be extrapolated to other specimens, such as complexes of larger size, and different data collection parameters, such as higher defocus.

Supplementary References

1 Rosenthal, P. B. & Henderson, R. Optimal determination of particle orientation, absolute hand, and contrast loss in single-particle electron cryomicroscopy. *J. Mol. Biol.* **333**, 721-745 (2003).

2 Andersen, G., Poterszman, A., Egly, J. M., Moras, D. & Thierry, J. C. The crystal structure of human cyclin H. *FEBS Lett.* **397**, 65-69 (1996).

3 Tsytlonok, M. *et al.* Dynamic anticipation by Cdk2/Cyclin A-bound p27 mediates signal integration in cell cycle regulation. *Nat. Commun.* **10**, 1676 (2019).

4 Pintilie, G. *et al.* Measurement of atom resolvability in cryo-EM maps with Q-scores. *Nat. Meth.* **17**, 328-334 (2020).

5 Hazel, P. *et al.* Inhibitor Selectivity for Cyclin-Dependent Kinase 7: A Structural, Thermodynamic, and Modelling Study. *ChemMedChem* **12**, 372-380 (2017).

6 Ali, S. *et al.* The development of a selective cyclin-dependent kinase inhibitor that shows antitumor activity. *Cancer Res.* **69**, 6208-6215 (2009).

7 Heathcote, D. A. *et al.* A Novel Pyrazolo[1,5-a]pyrimidine Is a Potent Inhibitor of Cyclin-Dependent Protein Kinases 1, 2, and 9, Which Demonstrates Antitumor Effects in Human Tumor Xenografts Following Oral Administration. *J. Med. Chem.* **53**, 8508-8522 (2010).

8 Bondke, A. *et al.* Pyrazolo[1,5-a]pyrimidine-5,7-diamine compounds as cdk inhibitors and their therapeutic use. U.S. patent US 9932344 B2 (2016).

9 Greber, B. J., Remis, J., Ali, S. & Nogales, E. 2.5Å-resolution structure of the human CDK-activating kinase bound to the clinical inhibitor ICEC0942. *Biophys J.* **120**, 677-686 (2021).

10 Herzik, M. A., Wu, M. & Lander, G. C. High-resolution structure determination of sub-100 kDa complexes using conventional cryo-EM. *Nat. Commun.* **10**, 1032 (2019).

11 Thangaratnarajah, C., Rheinberger, J. & Paulino, C. Cryo-EM studies of membrane proteins at 200 keV. *Curr. Opin. Struct. Biol.* **76**, 102440 (2022).

12 Wu, M., Lander, G. C. & Herzik, M. A. Sub-2 Angstrom resolution structure determination using single-particle cryo-EM at 200 keV. *J. Struct. Biol.: X* **4**, 100020 (2020).

13 Merk, A. *et al.* 1.8 Å resolution structure of β-galactosidase with a 200 kV CRYO ARM electron microscope. *IUCrJ* **7**, 639-643 (2020).

14 Punjani, A., Rubinstein, J. L., Fleet, D. J. & Brubaker, M. A. cryoSPARC: algorithms for rapid unsupervised cryo-EM structure determination. *Nat. Meth.* **14**, 290-296 (2017).

15 Kimanius, D., Dong, L., Sharov, G., Nakane, T. & Scheres, S. H. W. New tools for automated cryo-EM single-particle analysis in RELION-4.0. *Biochem J.* **478**, 4169-4185 (2021).

16 Merk, A. *et al.* Breaking Cryo-EM Resolution Barriers to Facilitate Drug Discovery. *Cell* **165**, 1698-1707 (2016).

17 Fukuda, Y., Laugks, U., Lučić, V., Baumeister, W. & Danev, R. Electron cryotomography of vitrified cells with a Volta phase plate. *J. Struct. Biol.* **190**, 143-154 (2015).

18 Nakane, T. *et al.* Single-particle cryo-EM at atomic resolution. *Nature* **587**, 152-156 (2020).

19 Yip, K. M., Fischer, N., Paknia, E., Chari, A. & Stark, H. Atomic-resolution protein structure determination by cryo-EM. *Nature* **587**, 157-161 (2020).
