# Supplementary material for: High-resolution cryo-EM of the human CDK-activating kinase for structure-based drug design
Source: Nat Commun. 2024 Mar 13;15:2265. doi: 10.1038/s41467-024-46375-9 (PMC10937634; doi:10.1038/s41467-024-46375-9)
Supplement: Supplementary file 2 — Reporting Summary [file 41467_2024_46375_MOESM2_ESM.pdf]

nature portfolio

Corresponding author(s): Basil J. Greber, Abhay Kotecha, Simak Ali

Last updated by author(s): Dec 18, 2023

Reporting Summary

Nature Portfolio wishes to improve the reproducibility of the work that we publish. This form provides structure for consistency and transparency in reporting. For further information on Nature Portfolio policies, see our Editorial Policies and the Editorial Policy Checklist.

Statistics

For all statistical analyses, confirm that the following items are present in the figure legend, table legend, main text, or Methods section.

n/a	Confirmed	
☐	☒	The exact sample size (*n*) for each experimental group/condition, given as a discrete number and unit of measurement
☐	☒	A statement on whether measurements were taken from distinct samples or whether the same sample was measured repeatedly
☒	☐	The statistical test(s) used AND whether they are one- or two-sided<br>*Only common tests should be described solely by name; describe more complex techniques in the Methods section.*
☒	☐	A description of all covariates tested
☒	☐	A description of any assumptions or corrections, such as tests of normality and adjustment for multiple comparisons
☐	☒	A full description of the statistical parameters including central tendency (e.g. means) or other basic estimates (e.g. regression coefficient) AND variation (e.g. standard deviation) or associated estimates of uncertainty (e.g. confidence intervals)
☒	☐	For null hypothesis testing, the test statistic (e.g. *F*, *t*, *r*) with confidence intervals, effect sizes, degrees of freedom and *P* value noted<br>*Give P values as exact values whenever suitable.*
☒	☐	For Bayesian analysis, information on the choice of priors and Markov chain Monte Carlo settings
☒	☐	For hierarchical and complex designs, identification of the appropriate level for tests and full reporting of outcomes
☒	☐	Estimates of effect sizes (e.g. Cohen's *d*, Pearson's *r*), indicating how they were calculated

Our web collection on statistics for biologists contains articles on many of the points above.

Software and code

Policy information about availability of computer code

Data collection	Thermo Fisher Scientific EPU and EPU multi-grid versions 2.14 (Glacios) and 3.1 (Krios) as well as Sherpa 2.0 were used for cryo-EM data collection.
Data analysis	Cryo-EM data were analysed using cryoSPARC (version 3.3.1-4.1.1) and RELION (version 4.0 beta 4.0-beta). Atomic models were built in COOT version 0.9.6. Atomic coordinates were refined using PHENIX (versions 1.20, 1.21), SCHRODINGER (version 2021-4), and SERVALCAT (0.2.122). Enzyme inhibition data were analysed in GraphPad PRISM (versions 9 and 10). Map and model visualisation, interpretation, and preparation of figures were performed in UCSF ChimeraX (version 1.2.5) and PyMOL (2.5.2-2.5.5). LC-MS raw data analysis was performed using Agilent MassHunter Qualitative Analysis, version B.07.00.

For manuscripts utilizing custom algorithms or software that are central to the research but not yet described in published literature, software must be made available to editors and reviewers. We strongly encourage code deposition in a community repository (e.g. GitHub). See the Nature Portfolio guidelines for submitting code & software for further information.

Data

Policy information about availability of data

All manuscripts must include a data availability statement. This statement should provide the following information, where applicable:

- Accession codes, unique identifiers, or web links for publicly available datasets
- A description of any restrictions on data availability
- For clinical datasets or third party data, please ensure that the statement adheres to our policy

Cryo-EM maps generated from 1-hour Glacios screening in this study have been deposited to the Electron Microscopy Data Bank (EMDB) with accession codes EMD-17470 [https://www.ebi.ac.uk/pdbe/entry/emdb/EMD-17470], EMD-17471 [https://www.ebi.ac.uk/pdbe/entry/emdb/EMD-17471], EMD-17472 [https://www.ebi.ac.uk/pdbe/entry/emdb/EMD-17472], EMD-17473 [https://www.ebi.ac.uk/pdbe/entry/emdb/EMD-17473], EMD-17474 [https://www.ebi.ac.uk/pdbe/entry/emdb/EMD-17474], EMD-17475 [https://www.ebi.ac.uk/pdbe/entry/emdb/EMD-17475], EMD-17476 [https://www.ebi.ac.uk/pdbe/entry/emdb/EMD-17476], EMD-17477 [https://www.ebi.ac.uk/pdbe/entry/emdb/EMD-17477], EMD-17478 [https://www.ebi.ac.uk/pdbe/entry/emdb/EMD-17478], EMD-17479 [https://www.ebi.ac.uk/pdbe/entry/emdb/EMD-17479], EMD-17480 [https://www.ebi.ac.uk/pdbe/entry/emdb/EMD-17480], EMD-17481 [https://www.ebi.ac.uk/pdbe/entry/emdb/EMD-17481], EMD-17482 [https://www.ebi.ac.uk/pdbe/entry/emdb/EMD-17482], EMD-17483 [https://www.ebi.ac.uk/pdbe/entry/emdb/EMD-17483], EMD-17484 [https://www.ebi.ac.uk/pdbe/entry/emdb/EMD-17484], EMD-17485 [https://www.ebi.ac.uk/pdbe/entry/emdb/EMD-17485], EMD-17486 [https://www.ebi.ac.uk/pdbe/entry/emdb/EMD-17486], EMD-17487 [https://www.ebi.ac.uk/pdbe/entry/emdb/EMD-17487], EMD-17488 [https://www.ebi.ac.uk/pdbe/entry/emdb/EMD-17488], EMD-17489 [https://www.ebi.ac.uk/pdbe/entry/emdb/EMD-17489], EMD-17490 [https://www.ebi.ac.uk/pdbe/entry/emdb/EMD-17490], EMD-17491 [https://www.ebi.ac.uk/pdbe/entry/emdb/EMD-17491], EMD-17492 [https://www.ebi.ac.uk/pdbe/entry/emdb/EMD-17492], EMD-17493 [https://www.ebi.ac.uk/pdbe/entry/emdb/EMD-17493], EMD-17494 [https://www.ebi.ac.uk/pdbe/entry/emdb/EMD-17494], and EMD-17495 [https://www.ebi.ac.uk/pdbe/entry/emdb/EMD-17495].

Cryo-EM maps resulting from 4-hour Glacios screening have been deposited to the EMDB with accession codes EMD-17496 [https://www.ebi.ac.uk/pdbe/entry/emdb/EMD-17496], EMD-17497 [https://www.ebi.ac.uk/pdbe/entry/emdb/EMD-17497], EMD-17498 [https://www.ebi.ac.uk/pdbe/entry/emdb/EMD-17498], EMD-17499 [https://www.ebi.ac.uk/pdbe/entry/emdb/EMD-17499], EMD-17500 [https://www.ebi.ac.uk/pdbe/entry/emdb/EMD-17500], EMD-17501 [https://www.ebi.ac.uk/pdbe/entry/emdb/EMD-17501], EMD-17502 [https://www.ebi.ac.uk/pdbe/entry/emdb/EMD-17502], EMD-17503 [https://www.ebi.ac.uk/pdbe/entry/emdb/EMD-17503], EMD-17504 [https://www.ebi.ac.uk/pdbe/entry/emdb/EMD-17504], EMD-17505 [https://www.ebi.ac.uk/pdbe/entry/emdb/EMD-17505], EMD-17506 [https://www.ebi.ac.uk/pdbe/entry/emdb/EMD-17506], and EMD-17507 [https://www.ebi.ac.uk/pdbe/entry/emdb/EMD-17507].

High-resolution cryo-EM maps generated in this study have been deposited to the EMDB using accession codes EMD-17129 [https://www.ebi.ac.uk/pdbe/entry/emdb/EMD-17129], EMD-17508 [https://www.ebi.ac.uk/pdbe/entry/emdb/EMD-17508], EMD-17509 [https://www.ebi.ac.uk/pdbe/entry/emdb/EMD-17509], EMD-17510 [https://www.ebi.ac.uk/pdbe/entry/emdb/EMD-17510], EMD-17511 [https://www.ebi.ac.uk/pdbe/entry/emdb/EMD-17511], EMD-17512 [https://www.ebi.ac.uk/pdbe/entry/emdb/EMD-17512], EMD-17513 [https://www.ebi.ac.uk/pdbe/entry/emdb/EMD-17513], EMD-17514 [https://www.ebi.ac.uk/pdbe/entry/emdb/EMD-17514], EMD-17515 [https://www.ebi.ac.uk/pdbe/entry/emdb/EMD-17515], EMD-17516 [https://www.ebi.ac.uk/pdbe/entry/emdb/EMD-17516], EMD-17517 [https://www.ebi.ac.uk/pdbe/entry/emdb/EMD-17517], EMD-17518 [https://www.ebi.ac.uk/pdbe/entry/emdb/EMD-17518], EMD-17519 [https://www.ebi.ac.uk/pdbe/entry/emdb/EMD-17519], EMD-17520 [https://www.ebi.ac.uk/pdbe/entry/emdb/EMD-17520], EMD-17521 [https://www.ebi.ac.uk/pdbe/entry/emdb/EMD-17521], EMD-17522 [https://www.ebi.ac.uk/pdbe/entry/emdb/EMD-17522], EMD-17523 [https://www.ebi.ac.uk/pdbe/entry/emdb/EMD-17523], EMD-17536 [https://www.ebi.ac.uk/pdbe/entry/emdb/EMD-17536], and EMD-17754 [https://www.ebi.ac.uk/pdbe/entry/emdb/EMD-17754].

Atomic coordinates of high-resolution ligand-bound complexes generated in this study have been deposited to the Protein Data Bank (PDB) using identifiers PDB ID 8ORM [http://doi.org/10.2210/pdb8ORM /pdb], PDB ID 8P6V [http://doi.org/10.2210/pdb8P6V /pdb], PDB ID 8P6W [http://doi.org/10.2210/pdb8P6W/pdb], PDB ID 8P6X [http://doi.org/10.2210/pdb8P6X/pdb], PDB ID 8P6Y [http://doi.org/10.2210/pdb8P6Y/pdb], PDB ID 8P6Z [http://doi.org/10.2210/pdb8P6Z/pdb], PDB ID 8P70 [http://doi.org/10.2210/pdb8P70/pdb], PDB ID 8P71 [http://doi.org/10.2210/pdb8P71/pdb], PDB ID 8P72 [http://doi.org/10.2210/pdb8P72/pdb], PDB ID 8P73 [http://doi.org/10.2210/pdb8P73/pdb], PDB ID 8P74 [http://doi.org/10.2210/pdb8P74/pdb], PDB ID 8P75 [http://doi.org/10.2210/pdb8P75/pdb], PDB ID 8P76 [http://doi.org/10.2210/pdb8P76/pdb], PDB ID 8P77 [http://doi.org/10.2210/pdb8P77/pdb], PDB ID 8P78 [http://doi.org/10.2210/pdb8P78/pdb], PDB ID 8P79 [http://doi.org/10.2210/pdb8P79/pdb], PDB ID 8P7L [http://doi.org/10.2210/pdb8P7L/pdb], and PDB ID 8PLZ [http://doi.org/10.2210/pdb8PLZ/pdb] (as detailed in Supplementary Tables 5-11).

Electron micrograph movies for selected datasets have been deposited to the Electron Microscopy Public Image Archive (EMPIAR) with accession codes EMPIAR-11793 [http://doi.org/10.6019/EMPIAR-11793], EMPIAR-11799 [http://doi.org/10.6019/EMPIAR-11799], EMPIAR-11800 [http://doi.org/10.6019/EMPIAR-11800], EMPIAR-11807 [http://doi.org/10.6019/EMPIAR-11807], EMPIAR-11821 [http://doi.org/10.6019/EMPIAR-11821], and EMPIAR-11823 [http://doi.org/10.6019/EMPIAR-11823].

Atomic coordinate models used in this study are publicly available from the PDB under accession codes PDB ID 1JKW [http://doi.org/10.2210/pdb1JKW/pdb], PDB ID 3NS9 [http://doi.org/10.2210/pdb3NS9/pdb], PDB ID 4KD1 [http://doi.org/10.2210/pdb4KD1/pdb], PDB ID 5JQ5 [http://doi.org/10.2210/pdb5JQ5/pdb], PDB ID 5JQ8 [http://doi.org/10.2210/pdb5JQ8/pdb], PDB ID 6ATH [http://doi.org/10.2210/pdb6ATH/pdb], PDB ID 6Q4G [http://doi.org/10.2210/pdb6Q4G/pdb], PDB ID 6XD3 [http://doi.org/10.2210/pdb6XD3/pdb], PDB ID 6Z4X [http://doi.org/10.2210/pdb6Z4X/pdb].

Source data are provided with this paper.

Research involving human participants, their data, or biological material

Policy information about studies with human participants or human data. See also policy information about sex, gender (identity/presentation), and sexual orientation and race, ethnicity and racism.

Reporting on sex and gender	Our research did not involve human participants or animals.

Reporting on race, ethnicity, or other socially relevant groupings	Our research did not involve human participants or animals.
Population characteristics	Our research did not involve human participants or animals.
Recruitment	Our research did not involve human participants or animals.
Ethics oversight	Our research did not involve human participants or animals and did not require ethics approval.

Note that full information on the approval of the study protocol must also be provided in the manuscript.

Field-specific reporting

Please select the one below that is the best fit for your research. If you are not sure, read the appropriate sections before making your selection.

[x] Life sciences [ ] Behavioural & social sciences [ ] Ecological, evolutionary & environmental sciences

For a reference copy of the document with all sections, see nature.com/documents/nr-reporting-summary-flat.pdf

Life sciences study design

All studies must disclose on these points even when the disclosure is negative.

Sample size	Dataset sizes of cryo-EM experiments were determined according to workflow requirements (1-hour and 4-hour data collection) or according to reconstruction resolution targets (5,000-10,000 movies per dataset to reach approx. 2 Å resolution). Our analysis also shows that dataset sizes beyond 5,000-10,000 movies provide only modest resolution improvement. Enzyme inhibition measurements were performed with n=2 per concentration tested.
Data exclusions	Data exclusion during cryo-EM data collection and data processing was performed according to established standards in the field. Poor quality micrographs were excluded based on contamination with crystalline ice, high specimen motion leading to poor CTF fit estimates, and ice thickness. Cryo-EM particle images were classified using two- and three-dimensional classification algorithms to remove poor-quality particles.
Replication	During sample screening, we collected data from more than one (typically two) grids per specimen (inhibitor-bound CAK complex) with consistent results between grids, except for quality differences that can be explained by the stochasticity of the grid preparation process. High-resolution structures were determined from a single grid (exception - THZ1, where two grids were collected with highly consistent results even at very high resolution). The structure of the CAK (protein complex) was consistent across all data collection sessions, with minor differences such conformational changes in the protein depending on presence of specific ligands.
Randomization	The nature of this study does not require randomization because it does not involve a clinical trial or treatment allocation.
Blinding	The nature of this study does not require blinding because it does not involve a clinical trial or treatment allocation.

Reporting for specific materials, systems and methods

We require information from authors about some types of materials, experimental systems and methods used in many studies. Here, indicate whether each material, system or method listed is relevant to your study. If you are not sure if a list item applies to your research, read the appropriate section before selecting a response.

Materials & experimental systems

n/a	Involved in the study	
[x]	[ ]	Antibodies
[ ]	[x]	Eukaryotic cell lines
[x]	[ ]	Palaeontology and archaeology
[x]	[ ]	Animals and other organisms
[x]	[ ]	Clinical data
[x]	[ ]	Dual use research of concern
[x]	[ ]	Plants

Methods

n/a	Involved in the study	
[x]	[ ]	ChIP-seq
[x]	[ ]	Flow cytometry
[x]	[ ]	MRI-based neuroimaging

Eukaryotic cell lines

Policy information about cell lines and Sex and Gender in Research

Cell line source(s)	Our study used commercially available Spodoptera frugiperda Sf9 and Trichoplusia ni High5 insect cell lines purchased from Thermo Fisher (catalogue numbers 11496015 and B85502, respectively).
Authentication	Not tested.
Mycoplasma contamination	Not tested.
Commonly misidentified lines (See ICLAC register)	None.

Plants

Seed stocks	*Report on the source of all seed stocks or other plant material used. If applicable, state the seed stock centre and catalogue number. If plant specimens were collected from the field, describe the collection location, date and sampling procedures.*
Novel plant genotypes	*Describe the methods by which all novel plant genotypes were produced. This includes those generated by transgenic approaches, gene editing, chemical/radiation-based mutagenesis and hybridization. For transgenic lines, describe the transformation method, the number of independent lines analyzed and the generation upon which experiments were performed. For gene-edited lines, describe the editor used, the endogenous sequence targeted for editing, the targeting guide RNA sequence (if applicable) and how the editor was applied.*
Authentication	*Describe any authentication procedures for each seed stock used or novel genotype generated. Describe any experiments used to assess the effect of a mutation and, where applicable, how potential secondary effects (e.g. second site T-DNA insertions, mosiacism, off-target gene editing) were examined.*
